# Marine Organisms as Alkaloid Biosynthesizers of Potential Anti-Alzheimer Agents

**DOI:** 10.3390/md20010075

**Published:** 2022-01-15

**Authors:** Elisabete Lima, Jorge Medeiros

**Affiliations:** 1 Faculty of Science and Technology (FCT), Institute of Agricultural and Environmental Research and Technology (IITAA), University of Azores, 9500-321 Ponta Delgada, São Miguel, Açores, Portugal; elisabete.mc.lima@uac.pt; 2 Faculty of Science and Technology (FCT), Biotechnology Centre of Azores (CBA), University of Azores, 9500-321 Ponta Delgada, São Miguel, Açores, Portugal

**Keywords:** Alzheimer’s disease, alkaloid, marine organism, sponge, MTDL

## Abstract

The incidence of neurodegenerative diseases, such as Alzheimer’s disease (AD), increases continuously demanding the urgent development of anti-Alzheimer’s agents. Marine organisms (MO) have to create their own defenses due to the adverse environment where they live and so synthesize several classes of compounds, such as akaloids, to defend themselves. Therefore, the identification of marine natural products with neuroprotective effects is a necessity. Being that AD is not only a genetic but also an environmental complex disease, a treatment for AD remains to discover. As the major clinical indications (CI) of AD are extracellular plaques formed by β-amyloid (Aβ) protein, intracellular neurofibrillary tangles (NFTs) formed by hyper phosphorylated τ-protein, uncommon inflammatory response and neuron apoptosis and death caused by oxidative stress, alkaloids that may decrease CI, might be used against AD. Most of the alkalolids with those properties are derivatives of the amino acid tryptophan mainly with a planar indole scaffold. Certainly, alkaloids targeting more than one CI, multitarget-directed ligands (MTDL), have the potential to become a lead in AD treatment. Alkaloids to have a maximum of activity against CI, should be planar and contain halogens and amine quaternization.

## 1. Introduction

Today about 50 million persons are having dementia and it is foreseen that this number will increase 60% in 2030 and to 180% in 2050 [1]. Alzheimer’s disease (AD) is a form of dementia which, nowadays, is more and more usual. Deficient cholinergic function, memory loss, loss of intellectual function, neuronal death, and behavioral disorders are the symptoms of this disease. Some risk factors for AD have been found to be family medical history, elderliness, the apolipoprotein E (APOE) ε4 allele genotype, cardiovascular disease risk factors, way of living, and psychosocial factors [2]. There are two modes to develop AD. The first one is on people younger than 65 years old and so is called early onset of AD (EOAD). The development of EOAD is associated with genetic mutations, genes such as amyloid precursor protein (APP), presenilin 1 (PSEN1), and presenilin 2 (PSEN2) which are involved in the production of the β-amyloid (Aβ) peptides. The second one is on people older than 65 years old and is called late onset of AD (LOAD). More than 90% of cases diagnosed are associated with LOAD [3]. LOAD has been consistently associated with only one gene, APOE gene. The allele ε4 of APOE is a genetic risk factor [4,5], generating cognitive decline and cerebral amyloid in aged individuals [6]. APOE is produced in the neuroglial cells, astrocytes, and provides a way for the production of Aβ plaques and development of cerebral amyloid angiopathology [7]. Furthermore, APOEε4 has also been associated to tau pathology [8]. Nevertheless, up to 75% of APOEε4 homozygous carriers do not progress to AD and 50% of AD patients are not APOEε4 carriers [4,5]. Indeed, the genetic predisposition to LOAD and the contribution of the other risk factors remains unknown [3]. Though the mechanisms of action of these genes in AD pathogenesis have been studied extensively, the ones involved in the progression of AD remain unclear, suggesting that AD is not only a genetic but also environmental complex disease [9]. Owing to that complexity an efficacious treatment for AD remains to discover [10] and the action against it has concerned mostly the reduction of the clinical indications (CI) of the disease. The major CI of AD are extracellular plaques formed by Aβ protein, intracellular neurofibrillary tangles (NFTs) formed by hyper phosphorylated τ-protein, uncommon inflammatory response and neuron apoptosis and death caused by oxidative stress [11,12,13]. Aβ plaques may cause cell death as they interfere with the communication at synapses between neurons, while NFTs block the transport of essential molecules in neurons [2]. On the other hand, the composition of the Aβ plaques is mainly of Aβ peptides which come from the cleavage of APP. Indeed, APP can be cleaved by the amyloidogenic pathway involving the action of two enzymes β-secretase (BACE-1) and γ-secretase. The cleavage of APP is performed by BACE-1, resulting two fragments, β-APP, and a longer peptide with 99 amino acids. While β-APP is a soluble fragment, the 99 amino acids fragment is now cleaved by γ-secretase into amylogenic peptides of varying length, including Aβ40, Aβ42, and Aβ43 [14].

The imbalance between Aβ generation and its clearance causes disequilibrium and consequently cell death. So, one way to combat AD is preventing the appearance of the Aβ plaques.

τ-Protein is a protein that stabilizes the microtubules (MTs) but, when hyper-phosphorylated, it accumulates into tangles producing the NFTs [15,16]. Indeed, τ-protein holds up the MTs however, when hyper-phosphorylated, τ-protein aggregates itself and unties the MTs which become destabilized. MTs are very important for the cytoskeleton in eukaryotic cells. They take part in a number of important structural and regulatory functions. Structurally, MTs are formed by the polymerization of α- and β-tubulin heterodimers [17]. MTs are always vibrating alternating between growing and shrinking phases [18]. Due to this dynamism, MTs can change rapidly and produce several different arrangements within cells. As MTs may be formed by different isoforms of tubulin they have a dynamic nature and interact with associated proteins (MAPs), being very important in determining the morphology, stability, and their function in different cell types [19]. A failure of these tuned actions of MTs is related to the appearance of many neurodegenerative disorders including AD [20]. Therefore, the stabilization of MTs may potentially prevent AD progression.

Certainly, another way to prevent the disease is reducing hyper-phosphorylation of τ-protein, and, so, avoid MTs dysfunction. When τ-protein is hyper-phosphorylated it aggregates into paired helical and straight filaments that result in the formation of NFTs [21,22]. As the phosphorylation of τ-protein results from an equilibrium between τ-kinase and phosphatase activities, kinase inhibitors restrain the processes of aggregation and the formation of NFTs [23,24,25]. Thus, one of the key strategies to combat AD is the inhibition of the protein kinases used in the phosphorylation of τ-protein [26]. The main relevant protein kinases that interfere in τ-phosphorylation belong to τ-protein kinase and dual-specificity kinases sub-subfamilies. The τ-protein kinases involved are glycogen synthetase kinase-3 beta (GSK3β) and casein kinase 1 delta (CKlδ) whereas the dual-specificity kinases involved are dual-specificity tyrosine phosphorylation regulated kinase lA (DYRKlA) and cdc2-like kinase 1 (CLKl). Several key residues are conserved when comparing these protein kinases and so they show common binding patterns. However, while GSK3β hipper-phosphorylates τ-protein, also increasing the production of Aβ and mediating neuronal death, CKlδ hipper-phosphorylates τ-protein reducing binding of τ-protein to microtubules, DYRKlA phosphorylates APP and τ-proteins, increasing neuronal death and the formation of aggregates and CLKl phosphorylates the serine residues in serine/arginine-rich (SR) proteins [24,25,26,27,28,29,30,31,32,33,34,35,36]. GSK3β is considered the main enzyme involved in the formation of NFTs. It can be [10,14].

The development of AD may also be prevented inhibiting inflammatory response of microglial cells [37,38]. Microglia, the brain’s resident immune cells, under normal conditions, protect the brain from pathogens and help to maintain homeostasis of the tissues. They have an anti-inflammatory role and are involved in different functions as phagocytosis, steroid release, free radical reduction, and cellular repair [39]. When unreasonably insulted, microglia cells can transform themselves, modifying their shapes, enabling their phagocytic functions and releasing a variety of proinflammatory factors, such as nitric oxide (NO), tumor necrosis factor-α (TNF-α), interleukin-1 (IL-1), interleukin-6 (IL-6), reactive oxygen species (ROS), prostaglandin E2 (PGE2), and cyclooxygenase-2 (COX-2) [40,41,42]. The accumulation of proinflammatory factors results in damage and degeneration of the nearby neurons. Subsequently the damaged neurons release certain immune substances, which increase the inflammatory neurotoxicity and causes irreversible neuroinflammation [43,44,45]. So, a potential therapeutic strategy for combatting AD is the use of agents for inhibiting microglia response.

Cognitive decline in AD patients is associated with the deficiency of the brain neurotransmitter acetylcholine (ACh). However, upon action of the enzyme acetylcholinesterase (AChE), ACh breaks down and, by hydrolysis, gives acetate and choline. When that happens choline is up taken into the presynaptic neuron and carried out by the choline carriers and the signal transduction at the neuromuscular junction finishes rapidly [46]. Inhibition of AChE prevents the breakdown of ACh and subsequently increases its concentration and duration of action, which are considered to be clinically beneficial for AD patients. Thus, AChE inhibitors are widely used for the treatment of AD [47].

On the other hand, ACh binds to several receptors in the synaptic cleft. One of them, nicotinic ACh receptors (nAChRs) in the central nervous system, control the liberation of other neurotransmitters and are involved in cognitive processes and memory [47,48]. Thus, another strategy to combat AD is controlling nAChRs.

So, the disease pathology can progress through different pathways which can even be related. For instance, AChE accelerates the deposition of Aβ protein [49]. The AChE gets co-localized with Aβ deposits. The interactions between them produces AChE-Aβ complex, a very toxic substance, which in turn increases the intracellular calcium load and decreases mitochondrial membrane potential. The AChE-Aβ complex formation causes the neuronal cells death [50]. On the other hand, as mentioned above, AChE stimulates the protein kinase C (PKC) which inhibits GSK3β. Thus, the above mechanisms may work altogether through interaction between genetic, molecular, and cellular events [51].

Among several strategies that have been identified to combat AD, multi-target compounds represent an effective strategy for the treatment of this multifactorial disease [52,53,54]. For instance, neuroinflammation and cholinergic deficit are considered major contributing factors for AD. Thus, compounds which have activity against AChE and anti-inflammatory qualities are mutli-target compounds to combat AD.

Owing to a huge genetic diversity of organisms and an ecological and molecular diversity, oceans are a unique and rich source of active compounds. Marine organisms (MO) have to create their own defenses due to the adverse environment where they live with specific chemical and physical properties, such as, water salt concentrations, pressure, temperature (including extreme), light penetration, oxygen concentrations, radiation exposure, and ocean currents. Each marine environment, with their own specifities, causes that MO get adapted to that environment structurally (or morphologically), as well as, physiologically and behaviorally [55,56].

As there are several kingdoms of MO: Bacteria, Protozoans, Chromists (including Seaweeds), Fungi, Plants, and Animals including jellyfish, sponges, sea spiders, bryozoans, mussels, sea stars, fish, and whales, they can synthesize several classes of metabolites used to immobilize and capture prey and defend against predators. These molecules enclose the potential to become a lead in AD innovative drug discovery [56,57,58,59,60,61,62,63]. Some of those molecules, the alkaloids, are pharmacologically active secondary metabolites characterized for containing nitrogen in their chemical structures, with at least one nitrogen atom derived directly from an amino acid [64,65].

**Figure 1:**
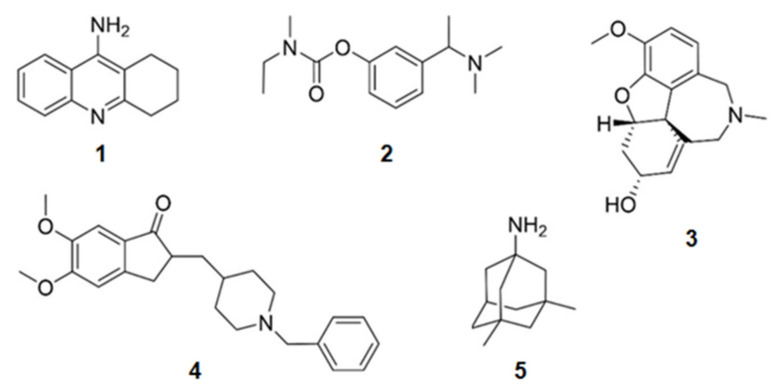
Chemical structures of the approved alkaloids for the treatment of Alzheimer’s disease. Adapted from [10].

Some compounds containing nitrogen in their structures have already been approved for the treatment of AD (Figure 1). Tacrine (**1**) (withdrawn from the market in 2012 due to adverse effects), donepezil (**2**), rivastigmine (**3**), and galantamine (**4**) were approved due to their activity on the inhibition of AChE. Menantine (**5**) is the other compound approved as a *N*-methyl-d-aspartate receptor antagonist. All of them are obtained synthetically. Although originally galantamine (**4**) was isolated from the terrestrial plant *Galanthus nivalis*, now it is produced by several synthetic routes.

It can be concluded that alkaloids from MO have a huge potential for being AD agents.

**Figure 2:**
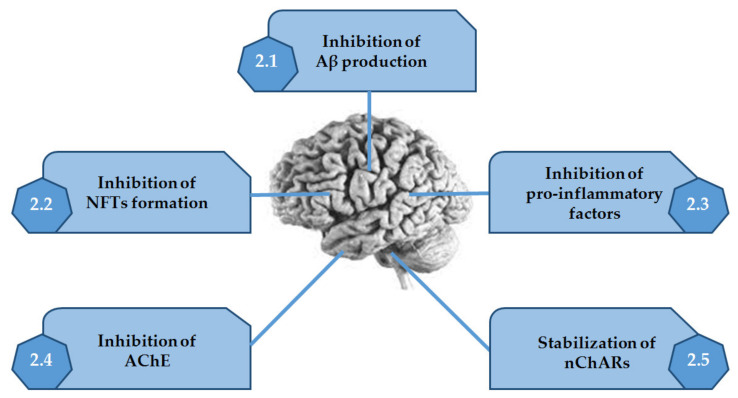
Activities of alkaloids discovered in marine organisms on the several mechanisms associated with Alzheimer’s disease. Adapted from [10].

Thus, in this review, are presented the several alkaloids discovered in MO, which are very active in the mechanisms described above (Figure 2 and Table 1).

## 2. Activities of Alkaloids Discovered in Marine Organisms

### 2.1. Inhibition of Aβ Production

Of the enzymes which are involved in the amyloidogenic pathway, β-secretase is the one which catalyzes the rate limiting step in the formation of amyloid plaques. So, then inhibition of this enzyme reduces the load of amyloid plaques in the neuronal cells by slowing or reversing the process [96].

#### 2.1.1. Derivatives of Tryptophan 

**Figure 3:**
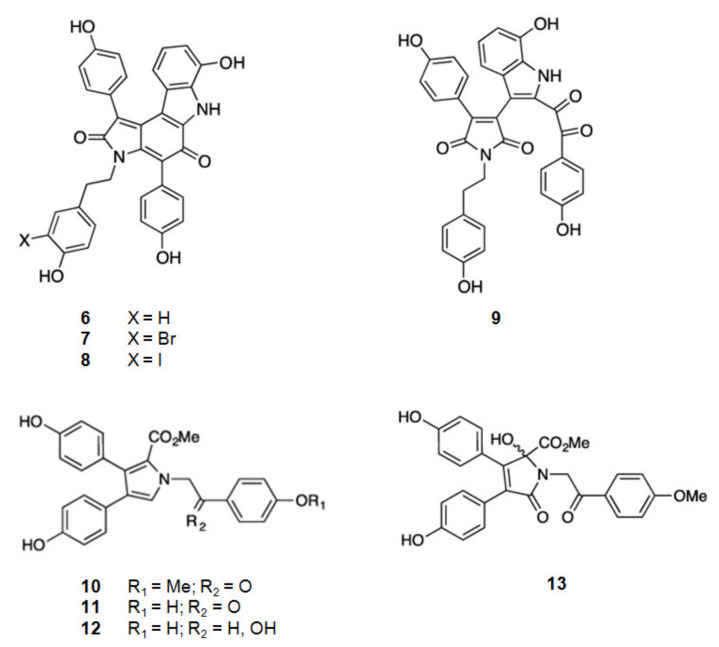
Structures of alkaloids from marine organisms that inhibit β-secretase.

From the Australian marine sponge *Ianthella* sp. a series of alkaloids in the class of the dictyodendrins were found (Figure 3). Dictyodendrins, pyrrolidone-containing alkaloids, are biosynthesized by the condensation between tryptophan and one or more substituted 4-hydroxyphenyl residues. Dictyodendrins F (**6**), H (**7**), I (**8**), and J (**9**) showed β-secretase inhibitory, being dictyodendrin H (**7**) the most potent one [66,67,97].

#### 2.1.2. Derivatives of Tyrosine

From the same marine sponge, *Ianthella* sp., other series of compounds were isolated, the lamellarins and the ianthellidones [66,67] (Figure 3). The lamellarins, pyrrole-containing alkaloids, were reported as lamellarins O (**10**), O1 (**11**), and O2 (**12**). As ianthellidons, pyrrolidone containing molecules, was only reported ianthellidone F (**13**). The biosynthesis of lamellarins is seen as a condensation between tyrosine and one or more substituted 4-hydroxyphenyl residues. The ianthellidones are viewed as oxygen addition adducts of lamellarins. The lamellarins and ianthellidone F (**13**) showed reduction of β-secretase activity, being lamellarin O1 (**6**) the most potent.

### 2.2. Inhibition of NFTs Formation

#### 2.2.1. Inhibition of GSK3β

##### Derivatives of Tryptophan

**Figure 4:**
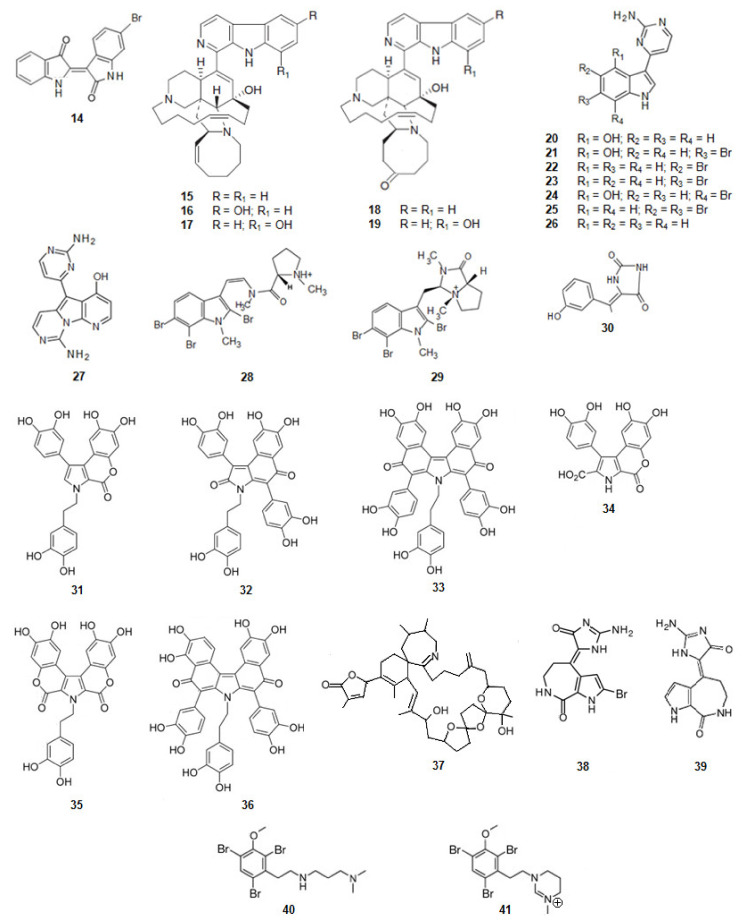
Structures of alkaloids from marine organisms that inhibit the kinase GSK3β.

6-Bromoindirubin (**14**) (Figure 4) was found from the Mediterranean mollusca *Hexaplex trunculus* and reported as a selective inhibitor of GSK3β [68]. It is a bis-indole alkaloid.

*Acanthostrongylophora* sp., a sponge from the Pacific Ocean, biosynthesizes manzamine alkaloids [98,99,100] (Figure 4). Manzamine A (**15**), E (**18**), F (**19**), Y (**16**), and 8-hydroxymanzamine A (**17**) are moderate, but significant inhibitors of human GSK3β activity. In a cell-based assay most of the compounds, but especially manzamine A (**15**) and 8-hydroxymanzamine A (**17**), showed a strong ability to inhibit tau phosphorylation within cells. From these data it can be concluded that the activity of GSK3β of the manzamine alkaloids is reduced by the hydrogenation of the double bond at C-32/C-33 and oxidation of C-31 to the ketone. It should also be mentioned that, when the manzamine alkaloids were evaluated in a series of assays relevant to nervous system function and pathology, they did not show any effect on AChE or β-amyloid cleaving enzyme (β-secretase) or any significant ability to protect human neuroblastoma SH-SY5Y cells against oxidative stress-induced cell death [69]. It can be concluded that this complex scaffold can be used to inhibit the hyper-phosphorylation of τ-protein and the appearance of intracellular NFTs but has no effect on the other clinical characteristics of AD.

From tunicate *Aplidium meridianum* and another tunicate in the genus of *Synoicum,* from the Antarctic, a series of indole alkaloids connected to an aminopyrimidine ring, were extracted, the meridianins [101,102,103] (Figure 4). Meridianins A (**20**), B (**21**), C (**22**), D (**23**), E (**24**), F (**25**), and G (**26**) are inhibitors of GSK3β [70,101]. Meridianin B (**21**) was found to be the most potent inhibitor of GSK3β among this series of compounds. The other meridianins; except meridianin G (**26**), were also very active as GSK3β inhibitors.

Meridianins are able to bind to all the six regions of protein kinases with a different binding strength depending on their chemical structure. They establish hydrophobic interactions, in the N-terminal lobe of the protein kinase, with the aminopyrimidine ring, revealing that this part of the molecule is important for optimal interactions [101,104]. Being small molecules, they can interact with the phosphate binding groove, located in the C-terminal lobe of the protein kinase. This is a rich polar region that consequently can create intermolecular interactions [105].

Variolin B (**27**) (Figure 4) is an alkaloid, also with an aminopyrimidine ring, and also inhibitor of GSK3β. It was extracted from *Kirkpatrickia vaialosa*, a sponge from the Antarctic Ocean [71,106].

Kororamides A (**28**) and B (**29**) (Figure 4) are tribrominated tryptophan derivatives, with an indole scaffold bearing an internal carbamoyl group, extracted from the bryozoan *Amathia tortuosa* [107,108]. Docking calculations and molecular dynamics (MD) simulations indicate that both compounds could bind to the ATP binding pocket of GSK3β, thus theoretically acting as ATP competitive inhibitors. Binding energies obtained after docking and MD simulations showed that kororamide A (**28**) presented better energies when bound against GSK3β, suggesting that the cyclization of the internal carbamoyl group decreases the inhibition of the ATP binding pocket [72,109,110].

##### Derivatives of Tyrosine

From a sponge *Hemimycale arabica* in the Red Sea, an alkaloid was extracted, (Z)-5-(4-hydroxybenzylidene)-hydantoin (**30**) (Figure 4). It binds directly to GSK3β inhibiting its activity [73].

##### Derivatives of 3,4-Dihydroxyphenylalanine (DOPA)

Ningalins, another class of compounds, derivatives from DOPA, contain several catechol groups (Figure 4). They were isolated from an Australian ascidian of the genus *Didemnum* [74,111]. While ningalins C (**32**), D (**33**), and G (**36**) present strong activity as GSK3β inhibitors, ningalins B (**31**), E (**34**), and F (**35**) presented moderate activity [74], suggesting that the number of catechol structures is determinant on the activity presented by this class of alkaloids.

##### Derivatives of Glycine

The dinoflagellate *Alexandrium ostenfeldii* biosynthesizes and accumulates in shellfish the spirolide, 13-desmethyl spirolide C (**37**) (Figure 4) [112]. It reduces the activity of GSK3β [75].

##### Derivatives of Proline

From the sponges *Axinella verrucosa* and *Acanthella aurantiaca* the alkaloid hymenaldisine (**38**) (Figure 4) was isolated [113]. The compound showed to be a potent GSK3β inhibitor with the IC_50_ value of 10 nM. That activity results from the competition of hymenaldisine (**38**) with ATP for binding to GSK3β. Hymenaldisine (**38**) prevents the hyperphosphorylation of tau protein both in vitro and in vivo [114]. It should be mentioned that the presence of the bromine atom at C-2 does not decreases the activity of GSK3β. Debromohymenialdisine (**39**) (Figure 4), a naturally occurring analog of hymenaldisine with the lack of the bromine atom at C-2 was isolated from the sponge *Phakellia flabellate* [115]. Likewise, hymenaldisine (**38**) and debromohymenialdisine (**39**) also inhibited the activity of GSK3β [76].

##### Derivatives of Phenylalanine

Another family of compounds extracted from the bryozoan, genus *Amathia*, are the convolutamines (Figure 4). Convolutamines I (**40**) and J (**41**) are halogenated heterocyclic compounds as other known kinase inhibitors. Thus, docking calculations and MD simulations were carried out to evaluate if convolutamines I–J could bind to GSK3β. The results indicate that both the compounds could bind to the ATP binding pocket of GSK3β [72,109,110].

#### 2.2.2. Inhibition of CKlδ 

Meridianins (**22**–**25**), derivatives of tryptophan, mentioned above as inhibitors of GSK3β, are also CKlδ inhibitors [101,104]. As explained above, several key residues are conserved when comparing these two protein kinases and so they show a common binding pattern. Protein kinases, GSK3β and CK1δ, present 5 similar binding residues. For CK1δ the most active meridianins are B (**21**) and E (**24**). It seems that to increase the affinity of the ligand on this receptor, the aminopyrimidine moiety should be oriented towards the top of the hydrophobic pocket at the N-terminal region. It should be noted that all the active meridianins for both kinases, the meridianins B (**21**), C (**22**), D (**23**), E (**24**), and F (**25**), contain bromine atoms, i.e., they are polar molecules and so can establish strong molecular interactions in the phosphate binding groove. As meridianin B (**21**) and E (**24**) contain, also, a hydroxy group it can also have other strong intermolecular interactions such as hydrogen bonds.

Other alkaloids, derivatives of tryptophan, which are common inhibitors of GSK3β and CK1δ, due to the similar binding pattern of both protein kinases, are variolin B (**27**) [71], kororamide A (**28**) and B (**29**) [71,72,107,108,109], as well as, convolutamine I (**51**) and J (**52**), derivatives of phenylalanine [72,109,110]. Hymenaldisine (**38**) and debromohymenialdisine (**39**) are two other alkaloids, derivatives of proline, that inhibit both protein kinases.

#### 2.2.3. Inhibition of DyrklA

**Figure 5:**
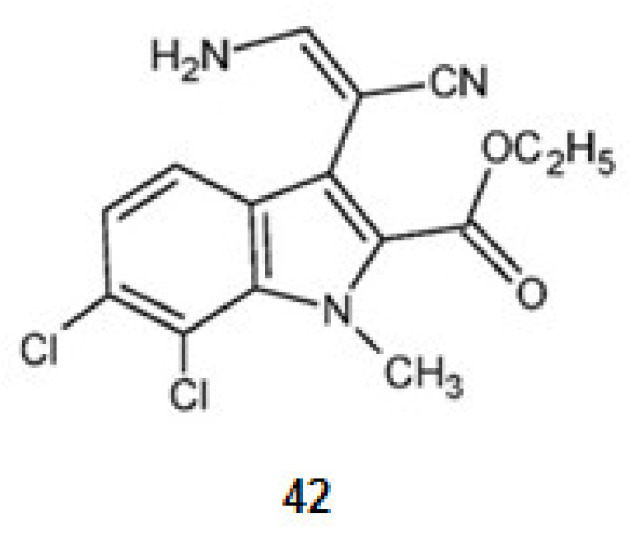
Structures of KH-CB 19 from *Dichothrixbaueriana* that inhibit the kinase DyrklA.

KH-CB 19, dichloroindolylenaminonitrile (**42**) (Figure 5), a derivative of tryptophan, extracted from the blue-green algae *Dichothrixbaueriana* showed activity as DyrklA inhibitor [109]. This compound is in pre-clinical trials [1].

As mentioned above, kororamides A (**28**) and B (**29**) are tribrominated tryptophan derivatives, with an indole scaffold bearing an internal carbamoyl group, extracted from the bryozoan *Amathia tortuosa* [116,117]. By docking and MD simulations, kororamide A–B were compared. Kororamide A (**28**) shows better energies against DyrklA [72].

#### 2.2.4. Inhibition of CLKl

KH-CB 19, dichloroindolylenaminonitrile (**42**), a derivative of tryptophan is a potent inhibitor not only for DyrklA, as already mentioned, but also for CLKl [77].

When comparing kororamide A–B and convolutamine I–J by docking and MD simulations kororamide A (**28**) presented better energies against CLK1 [109].

#### 2.2.5. MT-Stabilizing

**Figure 6:**
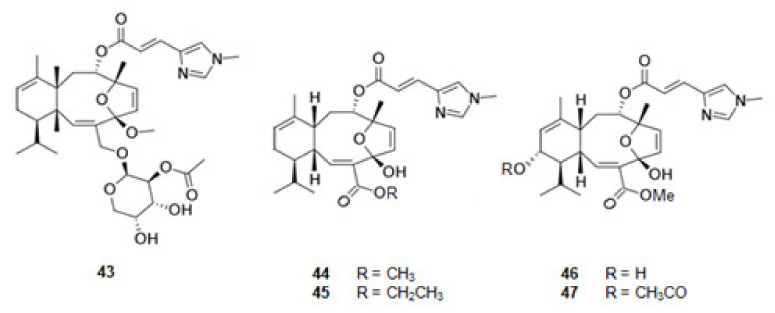
Structures of alkaloids from marine organisms that stabilize the microtubules.

Eleutherobin (**43**) [117,118] (Figure 6), a derivative of l-histidine, was extracted from the coral *Eleutherobia* sp. Sarcodyctins A (**44**), B (**45**), C (**46**), and D (**47**) (Figure 6) were extracted from the coral *Sarcodictyon roseum* [118,119]. They are alkaloids with the same scaffold and promote MT-stabilization [117,120] by interaction with β-tubulin at the taxane binding site [20].

### 2.3. Inhibition of Pro-Inflammatory Factors 

**Figure 7:**
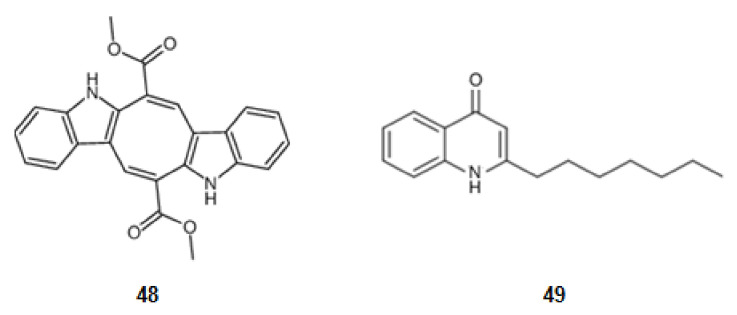
Structures of alkaloids from marine organisms that inhibit pro-inflammatory factors.

From a seaweed in the family *Caulerpaceae, Caulerpa racemosa* (Forsskål) J. Agardh, from the Northeast of Brazil, was extracted caulerpin (**48**) (Figure 7), a derivative of tryptophan. This bisindole alkaloid inhibits cyclooxygenase (COX), a key enzyme in inflammatory processes [78,121].

A secondary metabolite from a bacterial strain, *Pseudoalteromonas* sp., pseudane-VII (4-hydroxy-2-alkylquinoline) (**49**) (Figure 7), derivative of anthranilic acid, inhibits the LPS-stimulated NO, ROS production, and the expression of iNOS and COX-2 [79,122].

### 2.4. Inhibition of Acetylcholinesterase (AChE)

#### 2.4.1. Inhibitors from Bacteria

##### Derivatives of Tryptophan

**Figure 8:**
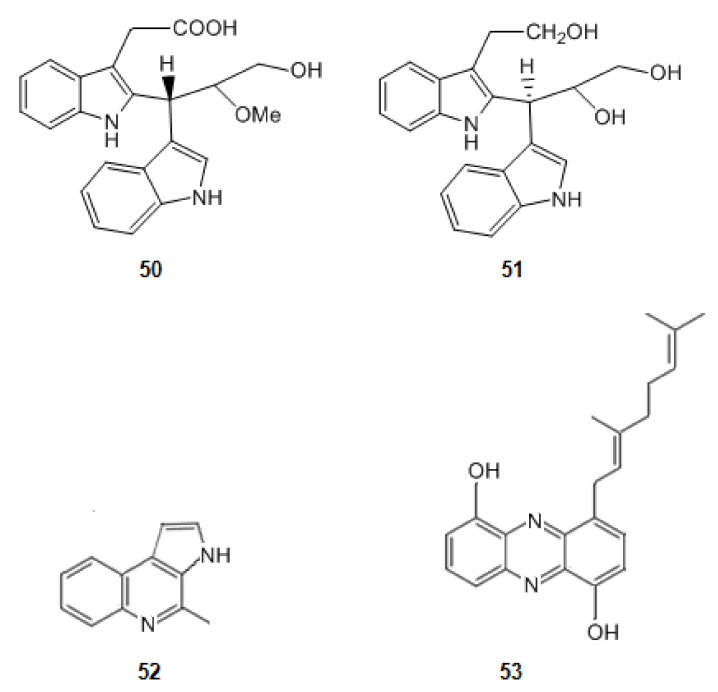
Structures of alkaloids from marine bacteria that inhibit acetylcholinesterase.

2-{2-[(1*R*)-3-Hydroxy-1-(1*H*-indol-3-yl)-2-methoxypropyl]-1*H*-indol-3-yl}acetic acid (**50**) and (3*S*)-3-[3-(2-hydroxyethyl)-1*H*-indol-2-yl]-3-(1*H*-indol-3-yl)propane-1,2-diol (**51**) (Figure 8) were isolated from a marine actinomycetes species, *Rubrobacter radiotolerans* [123]. They have a scaffold characterized by a dimeric indole nucleus. These alkaloids are moderately active as cholinesterase inhibitors. Indeed, nevertheless these molecules have two planar regions constituted by the two indole rings, they have not a corresponding extended aromatic system as, between them, there is a sp_3_ carbon, suggesting that the interaction between the esterase and the second indole group hinders the interaction of the active site of AChE and the first indole group.

When comparing the molecules **50** and **51** with marinoquinoline (**52**) (Figure 8), isolated from the bacterium *Rapidithrix thailandica*, in the south of Thailand, an increase in the inhibition of AChE is observed. Marinoquinoline (**52**) inhibits AChE with an IC value of 4.9 μM [49,80,124]. Indeed, the extensive aromatic ring system of it provides a huge planar π system which promotes π-π stacking interactions. Marinoquinoline interacts with AChE by participating in π-π stacking with Trp-84 [125].

##### Derivatives of Anthranilic Acid

A new phenazine derivative, geranylphenazinediol (**53**) (Figure 8) was produced by a *Streptomyces* sp. strain LB 173. This strain was obtained from a sediment sample of the brown algae *Saccharina latissima*, which was collected in the Kiel Fjord in Germany. This molecule also presents an extensive aromatic system. Geranylphenazinediol (**53**) inhibits AChE in micromolar range [81].

#### 2.4.2. Inhibitors from Fungi 

**Figure 9:**
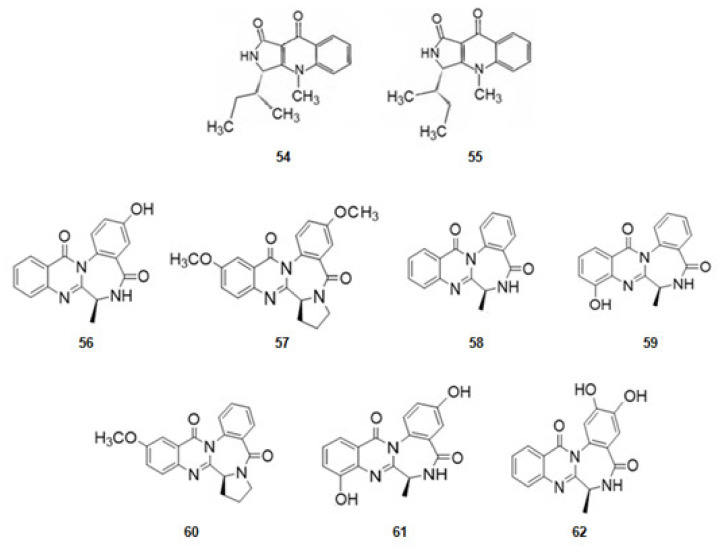
Structures of alkaloids from marine fungi that inhibit acetylcholinesterase.

From the sponge *Suberites domuncula* four isolates of fungal strains were obtained. From them quinolactacin A1 (**54**) and its optical isomer, A2 (**55**) (Figure 9), derivatives of tryptophan, were extracted. Quinolactacin A2 (**55**), just having a different configuration in one carbon, is much more active against AChE than A1 [82,126].

From the fungus *Aspergillusochraceus*, associated with corals, a series of several alkaloids with a quinazoline benzodiazepine scaffold, the circumdantins (Figure 9), derivatives of anthranilic acid, were extracted. Circumdantin C (**56**), D (**57**), F (**58**), G (**59**), H (**60**), I (**61**), and 2-hydroxycircumdantin C (**62**) were isolated. Circumdantin D (**57**) exhibited high inhibitory effect toward AChE and interference with pro-inflammatory response [83].

#### 2.4.3. Inhibitors from Animals: Jellyfish, Ascidian, and Molluscs

**Figure 10:**
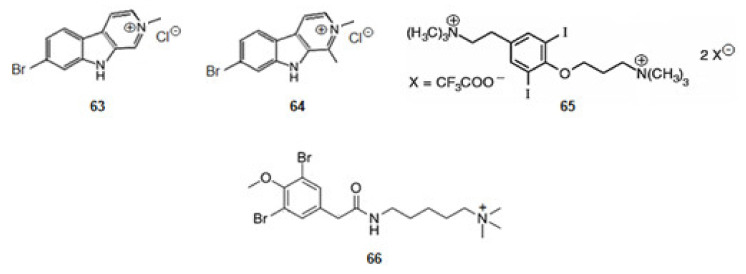
Structures of alkaloids from marine animals: ascidian and molluscs that inhibit acetylcholinesterase.

*Cnemidocarpa irene*, an ascidian, was collected from the Oshima-Kojima Islet in Japan. The irene-carbolines A (**63**) and B (**64**) (Figure 10) were extracted. The compounds, derivatives of tryptophan, exerted inhibition of AChE at sub-micromolar level [84].

*Turbo marmorata*, a snail, was collected from Okinawa in Japan. From the visceral extracts of it, turbotoxin A (**65**) (Figure 10), a derivative of tyrosine, was isolated [85]. The compound is active against AChE.

The ascidian *Synoicum pulmonaria* was collected off the coast of Tromsø, in Norway. Pulmonarin B (**66**) (Figure 10), a derivative of lysine, was extracted from the ascidian [86]. It is very active against AChE. From the results obtained for alkaloids turbotoxin A (**65**) and pulmonarin B (**66**) can be concluded that the cationic amine motif binds to the enzyme so that the alkaloid might exercise its effect [127].

#### 2.4.4. Inhibitors from Animals: Sponges and Corals

##### Derivatives of Tryptophan

**Figure 11:**
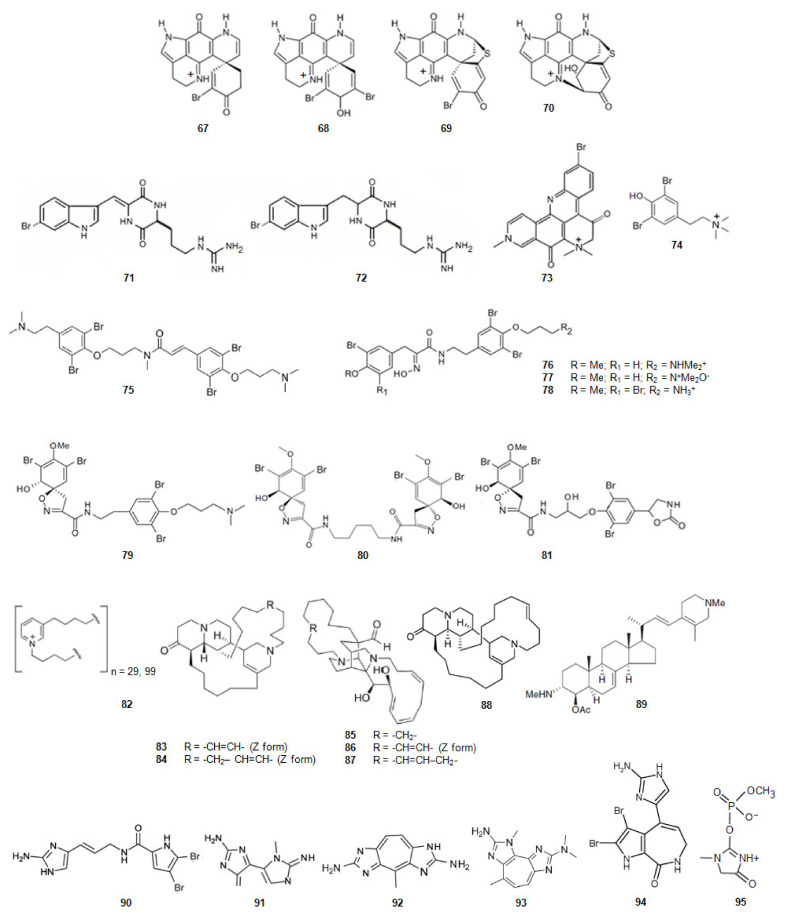
Structures of alkaloids from marine animals: sponges that inhibit acetylcholinesterase.

Sponges of the *Latrunculia* genus, dredged from the Antarctic Ocean, contained four discorhabdin alkaloids (Figure 11) [87]. From *Latrunculia biformis* (+)-discorhabdin G (**67**) [128] and (−)-3-dihydro-7,8-dehydrodiscorhabdin C (**68**) [129] were extracted whilst from *Latrunculia bocagei* (+)-discorhabdin B (**69**) [130] and (−)-discorhabdin L (**70**) [131] were isolated. (+)-Discorhabdin B (**69**) shows a higher activity against human recombinant AChE (hAChE) [87] suggesting that its three relevant H-bonds, in addition to a series of hydrophobic interactions due to the presence of the bromine and sulfur atoms.

The diketopiperazines barettin (**71**) and 8,9-dihydrobarettin (**72**) (Figure 11) [132,133] were extracted from the deep-sea sponge *Geodia barretti*, in Varangerfjorden, Norway. These compounds are brominated and derivatives simultaneously from two amino acids, tryptophan and ornithine. They were non-competitive inhibitors of AChE being 8,9-dihydrobarettin (**72**) more active. Comparing these results with the ones obtained for compounds 2-{2-[(1*R*)-3-Hydroxy-1-(1*H*-indol-3-yl)-2-methoxypropyl]-1*H*-indol-3-yl}acetic acid (**50**) and (3*S*)-3-[3-(2-hydroxyethyl)-1*H*-indol-2-yl]-3- (1*H*-indol-3-yl)propane-1,2-diol (**51**) it looks like a contradiction as, in this case, it is the most planar compound the less active. However, for compounds barettin (**71**) and 8,9-dihydrobarettin (**72**) the 2,5-diketopiperazine core is not so bulky as the indole group of alkaloids **50** and **51**. Compounds **71** and **72** also contain two carbonyl groups and two secondary amines which allow the establishment of very strong hydrogen bonding with AChE.

Petrosamine (**73**) (Figure 11), collected in Thailand, was extracted from a sponge of the genus *Petrosia* [88]. This alkaloid is a derivative from two amino acids, tryptophan and tyrosine. Petrosamine (**73**) shows strong AChE inhibitory activity. The most important interaction between petrosamine-AChE is due to the quaternary ammonium group of the alkaloid.

##### Derivatives of Phenylalanine or Tyrosine

The marine secondary metabolite stryphnusin (**74**) (Figure 11), a brominated phenethylamine, a derivative of phenylalanine, extracted from the sponge *Stryphnus fortis,* found in Norway, presents inhibitory activity against AChE [89]. 

From a sponge, *Suberea ianthelliformis*, collected off Nuku Hiva in French Polynesia, psammaplysene D (**75**) (Figure 11), a derivative of tyrosine was extracted [90]. It was found that it behaved as a mixed competitive/non-competitive inhibitor against AChE [80].

Aplysamine-2 (**76**) [90,134] and purpuramine J (**77**) (Figure 11) [135], also derivatives of tyrosine, were extracted from the sponge *Pseudoceratina* cf. *purpurea* from the Koh-Ha Islets, Thailand. While aplysamine-2 (**76**) inhibits AChE in a non-competitive manner, purpuramine J (**77**), differing from aplysamine-2 only by its rare N-oxide motif, is inactive. This fact suggests how important is the terminal dimethyl amine of aplysamine-2 for the inhibition of AChE. However, if that terminal is substituted by ammonium group, the inhibition of AChE is maintained. Indeed, aplysamine-4 (**78**) (Figure 11), which differs from aplysamine-2 (**76**) due to that group, isolated from the sponge *Psammaplysilla purpurea* [136] and *Verongida* sponge [137], is a potent inhibitor of AChE. If the terminal is the free amine (-NH_2_), obtained by alkali treatment of aplysamine-4, there is a significant reduction in the activity against AChE. On the other hand, purealidin Q (**79**) (Figure 11), a derivative of tyrosine, extracted from *Psammaplysilla purpurea*, is the most potent spiro-isoxazole containing inhibitor of AChE. Homoaerothioin (**80**) (Figure 11), also a derivative of tyrosine, extracted from the sponges *Verongia aerophoba* [138] and *Acanthodendrilla* sp. in Thailand [139], displays also low micromolar inhibitory activity against AChE. Fistularin 1 (**81**) (Figure 11), derivative of tyrosine, extracted from the sponges *Aplysina fistularis* forma *fulva* [140] and *Acanthodendrilla* sp. [139], is another alkaloid with the same spirocyclohexadienylisoxazoline structural feature of purealidin Q (**79**). It is also effective against AChE [139]. These facts show how important is the substitution of the N-terminal and the O-terminal of tyramine of the alkaloids with this scaffold. The spirocyclohexadienylisoxazoline group on the N-terminal and the N,N-dimethylaminopropyl group on the O-terminal show to be the best groups to improve the inhibition of AChE.

##### Derivatives of Nicotinic Acid

N-butyl(3-butyl-pyridinium) repeating subunits, polymerized head-to-tail, and existing as a mixture of two main polymers with molecular weights without counterion of about 5520 and 1890 (**82**) (Figure 11) was isolated from the sponge *Reniera sarai*. The monomer analogue of the inhibitor, N-butyl-3-butylpyridinium iodide has been synthesized. This molecule shows mixed reversible inhibition of AChE. The polymer act as AChE inhibitor and show an unusual inhibition pattern. Indeed, the AChE molecule contains several affinity binding sites where the polymer can bind. After the first binding has been accomplished, binding to other sites is favored leading to the formation of an irreversible enzyme-inhibitor complex [91]. 

A series of polycyclic diamine alkaloids, saraine 1 (**83**), saraine 3 (**84**), saraine A (**85**), saraine B (**86**), saraine C (**87**), and isosaraine 1 (**88**) (Figure 11) have been extracted from the sponge *Reniera sarai* [141,142,143,144,145,146] in the Mediterranean ocean. The mixture of saraines and isosaraine, inhibits the activity of AChE [92]. 

4-Acetoxy-plakinamine B (**89**) (Figure 11), collected from Thailand, from a sponge, *Corticium* sp., is a steroidal alkaloid [147]. This alkaloid reversibly inhibits AChE activity with the IC_50_ of 3.75 µM in a mixed-competitive mode [80].

##### Derivatives of Ornithine

Oroidin (**90**) (Figure 11), a dibromopyrrole compound, was extracted from the sponge *Agelas oroides* in the Mediterranean Sea [148,149]. The compound inhibits AChE [93]. 4,5-Dibromopyrrol-2-carboxylic acid corresponding to the left hand fragment of oroidin, was also isolated from *A. Oroides* is inactive against AChE [80].

Pseudozoanthoxanthin-like compound (PZT) (**91**) (Figure 11) was extracted from a coral *Parazoanthus axinellae* in the Mediterranean Sea, Italy. This coral is commonly known as the yellow cluster anemone. PZT is a competitive AChE inhibitor. The compound interacts with the aromatic residues lining the active site gorge. The alkaloid extinguishes the signal from the intrinsic tryptophan located in the gorge of AChE [150,151]. From *P. axinellae* was also isolated parazoanthoxanthin A (**92**) (Figure 11). It also inhibits AChE. Pseudozoanthoxanthin (**93**) (Figure 11) was extracted from a Pacific zoanthid of the *Gerardia* genus [152] and from an unidentified Mexican coral. Modeling studies suggest that this alkaloid would be capable of binding to both the catalytic and peripheral anionic sites of AChE [153]. The three alkaloids, oroidin (**90**), PZT (**91**) and parazoanthoxanthin A (**92**), and pseudozoanthoxanthin (**93**) have a huge extended aromatic system and are planar.

##### Derivatives of l-Proline

Stevensine (**94**) (Figure 11) extracted from a Micronesian sponge and from *Axinella verrucosa*, collected in the Mediterranean Sea, is a potent inhibitor of AChE [154]. It is a simultaneously derivative of the amino acids l-proline and histidine. It also presents a planar scaffold with an extended aromatic system.

##### Derivatives of Glycine

From the sponge *Ulosa ruetzleri*, collected off the coast of Bermuda, ulosantoin (**95**) (Figure 11), a phosphorylated hydantoin derivative, was extracted [155]. This compound inhibits AChE.

### 2.5. Stabilization of Nicotinic Acetylcholine Receptors (nAChRs)

When using the stabilization of the neuronal nicotinic ACh receptors (nACHrs) to control AD, compounds with mixed agonist/antagonist properties are required. Compounds that activate some α7 receptors and inhibit others and that afford the responses of other subtypes. Other compounds may stabilize nAChRs systems by activating one subtype and antagonizing another [156].

**Figure 12:**
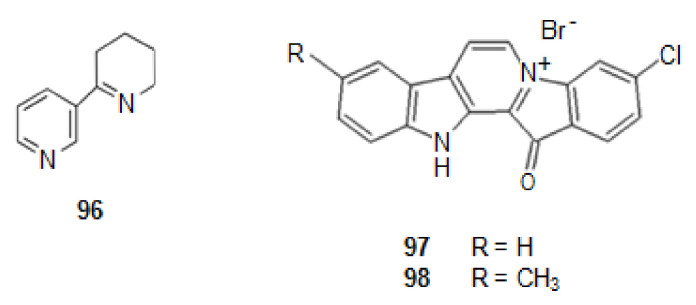
Structures of alkaloids from marine animals: sponges that stabilize nicotinic acetylcholine receptors.

Anabaseine (**96**) (Figure 12) is an example of alkaloid isolated from a marine ribbon-worm *Paranemertes peregrine* [157], which is a nonselective mixed agonist/antagonist for diverse types of nAChRs [94,158,159]. It is a simultaneously a derivative of nicotinic acid and lysine. GTS-21, a derivative of anabaseine is in phase II of clinical trials [10].

From a Fijian sponge *Fascaplysinopsis bergquist* sp. [10,95,160], a benzoyl-linked β-carboline alkaloid was isolated, fascaplysin (**97**) (Figure 12) [161]. This alkaloid shows P-gp induction activity. P-gp is an important member of the ATP-binding transporter which is related with AD. Indeed, when the level of P-gbp increases there is a decrease in the appearance of Aβ, as P-gp is a transporter for out of the brain [160].

Several derivatives of it were synthesized, chosen by molecular modeling studies. One of them, 9-methylfascaplysin (**98**) (Figure 12), showed to have P-gp induction activity.

This compound, also, shows great potential to activate antioxidant enzymes, and thus, to produce neuroprotective effects against ROS. It crosses the blood brain barrier (BBB) in mice, preventing cognitive damages through the inhibition of AChE in the hippocampus, without causing severe neurotoxicity. Thus, this molecule can preventive dysfunction, decrease neuroinflammation, and reduce tau hyper phosphorylation [161]. It is in clinical trials [10].

## 3. Conclusions

Nowadays 93 alkaloids, from marine organisms (MO), showed to be potential anti-Alzheimer agents: 47 (50.5%) were found in sponges, 7 (7.5%) in fungi, 9 (9.7%) in ascidian, 8 (8.6%) in corals, 5 (5.4%) in bacteria, 4 (4.3%) in bryozoan, and the other 13 (14.0%) were found in algae, dinoflagellates, mollusks, and worms. Half of the alkaloids active against AD are from sponges. This conclusion is explained as the result of the high content of opportunistic and symbiotic microorganisms on sponges [162,163]. Many of these alkaloids extracted from sponges present structures extremely different, however, most of them are derivatives of the amino acid tryptophan (42%), amino acid very planar with an extended aromatic system. Coincidentally, 40% of the very active alkaloids extracted from all the MO reported are also derivatives of tryptophan. Even, 65% of the alkaloids active, in all the mechanisms analyzed against AD, present in their structures an extended aromatic system suggesting that planarity and an extensive π system in the molecule is one of the requirements for alkaloids be active against AD. Indeed, a significant effect in inhibiting human GSK3β activity can be obtained using manzamine compounds. Manzamine A (**15**) and 8-hydroxymanzamine A (**17**), showed to be the most active inhibiting tau phosphorylation. This suggests that activity is due to the extended aromatic system of the β-carboline residue and, in these compounds, the double bond at C-32/C-33 is much more efficient for activity than the oxidation of C-31. However, manzamines did not show any effect on the inhibition of AChE or β-secretase, or the pro-inflammatory factors.

Meridianins have also been reported as potent inhibitors of GSK3β. Consisting of an indole framework connected to an aminopyrimidine ring, all the atoms are present with a hybridation sp_2_, and so the molecule is planar and has an extended π system. Meridianins can link to all the regions of the enzyme with different binding strength accordingly to their specific chemical structure. So, with this framework they bind to protein kinases in general, but owing to the substitution pattern of the indole ring, each one is more related with one of the protein kinases. For GSK3β, the most potent inhibitors are meridianins C (**22**), D (**23**), E (**24**), and F (**25**). They establish hydrophobic contacts with the aminopyrimidine ring. The meridianins C–F contain bromine atoms on the indole ring, suggesting that in bromine, the substitution of the aromatic indole group is important for the inhibition of GSK3β. For CK1δ the best inhibitors are meridianins C, D, and F. It seems that to increase the inhibition of this kinase C-4 of the indole ring should not be substituted. Variolin B (**27**), with a framework similar to meridianins, is also very active in the inhibition of GSK3β and CK1δ. Kororamide A (**28**) and B (**29**), also with a framework similar to meridianins, except for the aminopyrimidine ring, have also bromine atoms at the indole ring. Docking calculations and MD simulations show that kororamide A and B have activity against protein kinases, having kororamide A better energies for GSK3β, DyrklA and CLK1, suggesting that the opening of the aminopyrimidamine ring of meridianins and appearance of a pyrrolidinium ion, causes no effective change on the inhibition of kinases, mainly if the positive charge in the nitrogen atom is not hindered. These results show how the indole scaffold (planar scaffold) and the presence of halogen atoms are important in a molecule for the inhibition of GSK3β, CK1δ, DyrklA and CLK1. Marinoquinoline (**52**), a pyrrole quinolone derivative with an extended aromatic system, is a potent inhibitor of AChE. Other pyrrole derivatives, but lacking an extensive aromatic ring system are inactive, suggesting that inhibition produced by marinoquinoline was due to π-π stacking interactions only possible for planar molecules.

Irene-carbolines A (**63**) and B (**64**), β-carboline alkaloids, with an aromatic ring system, a cationic imine motif and one bromine atom linked to the phenyl group, are strong inhibitors, but many other β-carboline alkaloids, with molecules with the same characteristics, but not brominated, do not present that activity, suggesting, also, the need of the halogen substitution of the phenyl group. Pulmonarin B (**66**) with a O-methyl-dibrominated phenol group and a cationic quaternary amine motif and stryphnusin (**74**), a brominated phenethylamine, exhibit inhibitory activity against AChE suggesting that hydrophobic aryl substituents and amine quarternisation increase the inhibition of the enzyme, in part by binding to the cationic amine motif of the alkaloid.

Psammaplysene D (**75**) is an inhibitor against AChE. It should be noticed the importance of the trans-cinnamoyl group making the molecule planar at that part and the aromatic ring on the other side of the molecule. The two aromatic rings have two bromine atoms linked to each one.

Petrosamine (**73**), also with an extended aromatic ring system, shows strong AChE inhibitory activity. The interaction between petrosamine and the enzyme is mainly due to the cationic amine motif of the alkaloid.

The results reported here only concern the interaction of one alkaloid with the active site of one enzyme. However, AD has multiple pathogenic factors, as described. Thus, using more than one pharmacological approach can be highly advantageous [164,165] as AD is such a complex disease involving several mechanisms which may work altogether through interaction between genetic, molecular, and cellular events. One possible successful strategy might be multitarget-directed ligands (MTDL) that is, using a multitarget therapy. This therapy can be achieved by two ways. The first one, combination therapy, uses a drug cocktail, where each drug has an active component for the inhibition of one of the mechanisms of AD. This approach therapy is associated with high-risk drug–drug interactions. The second approach is referred to as MTDL where only one active ingredient is administered [166]. So, the risk of interaction between drugs is eliminated. Additionally, the prevision of pharmacokinetic and pharmacodynamics properties is simplified with a single agent. The MTDL strategy looks to be more advantageous [163]. Analyzing the scaffold of the several alkaloids isolated, which inhibit one of the mechanism of AD, it is concluded that all of those mechanisms are inhibited mostly by alkaloids with a planar core. For the inhibition of β-secretase 100% of the alkaloids isolated have a planar core. For the inhibition of Aβ plaques, NFTs, pro-inflammatory factors, AChE, and the stabilization of nChAR, the alkaloids isolated from MO have, respectively, 80, 84, 100, 61, and 100% structures with a planar core. So, as previously mentioned, alkaloids to have a maximum of activity for the several pathways of AD, should be planar. Even, for the several planar alkaloids isolated from MO, the inhibition of β-secretase is 50% from alkaloids derivatives of tryptophan. For the inhibition of Aβ plaques, NFTs, pro-inflammatory factors, AChE and the stabilization of nChAR, the planar alkaloids isolated from MO have, respectively, 100, 63, 100, 46, and 100% structures derivatives of the amino acid tryptophan. Thus, a MTDL, should be planar, with an extended aromatic system, at least like the one presented by an indole group (derivative of tryptophan). A planar MTDL should contain in their structure halogens, such as bromine, for the establishment of strong molecular interactions in the phosphate binding groove of several protein kinases, including GSK3β. A quaternary ammonium group on the planar MTDL is also required to interact with AChE. Some molecules have already showed activity, in more than one pathway.

Circumdantin D (**57**), with an aromatic ring system even more extended than the one of an indole group, inhibits AChE and interferes with pro-inflammatory response.

9-Methylfascaplysin (**12**), a fascaplysin derivative, inhibits the formation of Aβ plaques and AChE. It has also potential to activate antioxidant enzymes and thus, to produce neuroprotective effects against ROS. 9-Methylfascaplysin (**12**) contains in its scaffold an aromatic ring system also more extended than the one presented by the indole group and has a cationic iminium ion. Certainly, if this scaffold contained bromine atoms, it might be able to bind to the ATP binding pocket of GSK3β. Thus, fascaplysin derivatives can become a new class of potential multi-target drugs for AD.

Considering the alkaloids already isolated from MO, MTDL alkaloids inhibiting several pathways causing AD, should be identified. As all the pathways interact among them, the future research should focus not only on identifying alkaloids that are active against not only one, but several pathways.

## Figures and Tables

**Table 1:** Alkaloids from marine organisms with neurological activities and their mode of action.

Compound ^1^		Mechanism of Action	IC_50_ (μM)	Ref.
**6**	Dictyodendrin F	Inhibition of Aβ production	1.5	[66]
**7**	Dictyodendrin H		1.0	[66]
**8**	Dictyodendrin I		2.0	[66]
**9**	Dictyodendrin J		2.0	[66]
**10**	Lamellarin O		>10	[67]
**11**	Lamellarin O1		<10	[67]
**12**	Lamellarin O2		>10	[67]
**13**	Iianthellidone F		>10	[67]
**14**	6-Bromoindirubin	Inhibition of GSK3β	0.045	[68]
**15**	Manzamine A		10.0	[69]
**16**	Manzamine Y		<25	[69]
**17**	8-Hydroxymanzamine A		<25	[69]
**18**	Manzamine E		<25	[69]
**19**	Manzamine F		>25	[69]
**20**	Meridianin A	Inhibition of GSK3β	1.3	[70]
		Inhibition of CKlδ	NE	
**21**	Meridianin B	Inhibition of GSK3β	0.5	[70]
		Inhibition of CKlδ	1.0	
**22**	Meridianin C	Inhibition of GSK3β	2.0	[70]
		Inhibition of CKlδ	30.0	
**23**	Meridianin D	Inhibition of GSK3β	2.5	[70]
		Inhibition of CKlδ	100.0	
**24**	Meridianin E	Inhibition of GSK3β	2.5	[70]
		Inhibition of CKlδ	0.4	
**25**	Meridianin F	Inhibition of GSK3β	2.0	[70]
		Inhibition of CKlδ	NE	
**26**	Meridian G	Inhibition of GSK3β	350	[70]
		Inhibition of CKlδ	NE	
**27**	Variolin B	Inhibition of GSK3β	0.07	[71]
		Inhibition of CKlδ	0.005	
**28**	Kororamide A	Inhibition of GSK3β	NE	[72]
		Inhibition of CKlδ		
		Inhibition of DyrklA		
		Inhibition of CLK1		
**29**	Kororamide B	Inhibition of GSK3β	NE	[72]
		Inhibition of CKlδ		
**30**	(Z)-5-(4-Hydroxybenzylidene)-hydantoin	Inhibition of GSK3β	13.7	[73]
**31**	Ningalin B		0.8	[74]
**32**	Ningalin C		<0.2	[74]
**33**	Ningalin D		<0.2	[74]
**34**	Ningalin E		1.6	[74]
**35**	Ningalin F		3.1	[74]
**36**	Ningalin G		<0.5	[74]
**37**	13-Desmethyl spirolide C		NE	[75]
**38**	Hymenaldisine	Inhibition of GSK3β	0.07	[76]
		Inhibition of CKlδ	0.03	
**39**	Debromohymenialdisine	Inhibition of GSK3β	0.2	
		Inhibition of CKlδ	0.1	
**40**	Convolutamine I	Inhibition of GSK3β	NE	[72]
		Inhibition of CKlδ		
**41**	Convolutamine J	Inhibition of GSK3β		
		Inhibition of CKlδ		
**42**	KH-CB 19	Inhibition of DyrklA	0.06	[77]
		Inhibition of CLK1	0.02	
**43**	Eleutherobin	MT-stabilizing	NE	[20]
**44**	Sarcodyctin A			
**45**	Sarcodyctin B			
**46**	Sarcodyctin C			
**47**	Sarcodyctin D			
**48**	Caulerpin	Inhibition of pro-inflammatory factors	NE	[78]
**49**	Pseudane-VII			[79]
**50**	2-{2-[(1*R*)-3-Hydroxy-1-(1*H*-indol-3-yl)-2-methoxypropyl]-1*H*-indol-3-yl}acetic acid	Inhibition of AChE	11.8	[80]
**51**	(3*S*)-3-[3-(2-hydroxyethyl)-1*H*-indol-2-yl]-3-(1*H*-indol-3-yl)propane-1,2-diol		13.5	[80]
**52**	Marinoquinoline		4.9	[80]
**53**	Geranylphenazinediol		2.6	[81]
**34**	Quinolactacin A1		280	[82]
**55**	Quinolactacin A2		19.8	[82]
**56**	Circumdantin C		15.6	[83]
**57**	Circumdantin D		8.7	[83]
**58**	Circumdantin F		11.8	[83]
**59**	Circumdantin G		18.9	[83]
**60**	Circumdantin H		33.3	[83]
**61**	Circumdantin I		18.6	[83]
**62**	2-Hydroxycircumdantin C		16.5	[83]
**63**	Irene-carboline A		0.7	[84]
**64**	Irene-carboline B		0.5	[84]
**65**	Turbotoxin A		90.0	[85]
**66**	Pulmonarin B		20.0	[86]
**67**	(+)-Discorhabdin G	Inhibition of hAChE	116.0	[87]
**68**	(-)-3-Dihydro-7,8-dehydrodiscorhabdin C		152.0	
**69**	(+)-Discorhabdin B		49.4	
**70**	(-)-Discorhabdin L		158.2	
**71**	Barettin	Inhibition of AChE	36.0	[80]
**72**	8,9-Dihydrobarettin		29.0	[80]
**73**	Petrosamine		0.1	[88]
**74**	stryphnusin		232.0	[89]
**75**	Psammaplysene D		1.3	[90]
**76**	Aplysamine-2		1.3	[80]
**77**	Purpuramine J		NA	[80]
**78**	Aplysamine-4		16.0	[80]
**79**	Purealidin Q		1.2	[80]
**80**	Homoaerothioin	Inhibition of hAChE	4.5	[80]
**81**	Fistularin 1		47.5	
**82**	N-butyl(3-butyl-pyridinium)n	Inhibition of AChE	0.1	[91]
**83**	Saraine 1		6.4	[92]
**84**	Saraine 3		6.3	[92]
**85**	Saraine A		4.4	[92]
**86**	Saraine B		4.4	[92]
**87**	Saraine C		8.4	[92]
**88**	Isosaraine 1		10.7	[92]
**89**	4-Acetoxy-plakinamine B		3.8	[80]
**90**	Oroidin		<0.5	[93]
**91**	PZT		4.0	[80]
**92**	Parazoanthoxanthin A		26.0	[80]
**93**	Pseudozoanthoxanthin		12.2	[80]
**94**	Stevensine	Inhibition of hAChE	14.6	[80]
**95**	Ulosantoin	Inhibition of AChE	<0.1	[80]
**96**	Anabaseine	Stabilization of nAChRs	NE	[94]
**97**	Fascaplysin	Stabilization of nAChRs	NE	[95]
		Inhibition of AChE	1.49	[80]

^1^ Chemical structures of **6**–**13** (Section 2.1), **14**–**41** (Section 2.2.1), **42** (Section 2.2.3), **43**–**47** (Section 2.2.5), **48**–**49** (Section 2.3), **50**–**53** (Section 2.4.1), **54**–**62** (Section 2.4.2), **63**–**66** (Section 2.4.3), **67**–**95** (Section 2.4.4), and **96**–**97** (Section 2.5). hAChE—human recombinant acetylcholinesterase; NA—not active; NE—not evaluated.

## Data Availability

Not applicable.

## References

[1] Russo P., Kisialiou A., Lamonaca P., Moroni R., Prinzi G., Fini M. (2016). New drugs from marine organisms in Alzheimer’s disease. Mar. Drugs.

[2] Alzheimer’s Association (2019). Alzheimer’s disease facts and figures. Alzheimer’s Dement..

[3] Isik A.T. (2010). Late onset Alzheimer’s diseasae in older people. Clin. Interv. Aging.

[4] Van Cauwenberghe C., van Broeckhoven C., Sleegers K. (2016). The genetic landscape of Alzheimer disease: Clinical implications and perspectives. Genet. Med..

[5] Hauser P.S., Narayanaswami V., Ryan R.O. (2011). Apolipoprotein E: From lipid transport to neurobiology. Prog. Lipid Res..

[6] Liu C., Kanekiyo T., Xu H., Bu G. (2013). Apoliprotein E and Alzheimer disease: Risk, mechanisms and therapy. Nat. Ver. Neurol..

[7] Kim J., Basak J.M., Holtzman D.M. (2009). The role of apoliprotein E in Alzheimer’s disease. Neuron.

[8] Shi Y., Yamada K., Liddelow S.A., Smith S.T., Zhan L., Lun W., Tsai R.M., Spina S., Grinberg L.T., Rojas J.C. (2017). ApoEε4 markedly exacerbates tau-mediated neurodegeneration in a mouse mode tauopathy. Nature.

[9] Bekris L.M., Yu C., Bird T.D., Tsuang T.W. (2010). Genetics of Alzheimer’s disease. J. Geriatr. Psychiatry Neurol..

[10] Martins M., Silva R., MM Pinto M., Sousa E. (2020). Marine natural products, multitarget therapy and repurposed agents in Alzheimer’s disease. Pharmaceuticals.

[11] Anand P., Singh B., Singh N. (2012). A review on coumarins as acetylcholinesterase inhibitors for Alzheimer’s disease. Bioorg. Med. Chem..

[12] Macauley S.L., Holtzman D.M. (2015). Recent advances from the bench toward the bedside in Alzheimer’s disease. EBioMedicine.

[13] Takashima A. (2010). Tau aggregation is a therapeutic target for Alzheimer’s disease. Curr. Alzheimer Res..

[14] Coman H., Nemes B. (2017). New therapeutic targets in Alzheimer’s disease. Int. J. Gerontol..

[15] Cummings J.L., Tong G., Ballard C. (2019). Treatment combinatoirs for Alzheimer’s disease: Current and future pharmacotherapy options. J. Alzheimer’s Dis..

[16] Fish P.V., Steadman D., Bayle E.D., Whiting P. (2019). New approaches for the treatment of Alzheimer’s disease. Bioorgan. Med. Chem. Lett..

[17] Desai A., Mitchison T.J. (1997). Microtubule polymerization dynamics. Annu. Rev. Cell Dev. Biol..

[18] Mitchison T., Kirschner M. (1984). Dynamic instability of microtubule growth. Nature.

[19] Ballatore C., Brunden K.R., Huryn D.M., Trojanowski J.Q., Lee V.M.-Y., Smith A.B. (2012). Microtubule stabilizing agents as potential treatment for Alzheimer’s disease and related neurodegenerative tauopathies. J. Med. Chem..

[20] White J.A., Banerjee R., Gunawardena S. (2016). Axonal transport and neurodegeneration: How marine drugs can be used for the development of therapeutics. Mar. Drugs.

[21] Kosik K.S., Joachim C.L., Selkoe D.J. (1986). Microtubule-associated protein tau (tau) is a major antigenic component of paired helical filaments in Alzheimer disease. Proc. Natl. Acad. Sci. USA.

[22] Naini S., Soussi-Yanicostas N. (2015). Tau hyperphosphorylation and oxidative stress, a critical vicious circle in neurodegenerative tauopathies?. Oxidative Med. Cell. Longev..

[23] Kolarova M., García-Sierra F., Bartos A., Ricny J., Ripava D., Ripava D. (2012). Structure and pathology of tau protein in Alzheimer disease. Int. J. Alzheimers Dis..

[24] Martin L., Latypova X., Wilson C.M., Magnaudeix A., Perrin M.-L., Yardin C., Terra F. (2013). Tau protein kinases: Involvement in Alzheimer’s disease. Ageing Res. Rev..

[25] Citron M. (2010). Alzheimer’s disease: Strategies for disease modification. Nat. Rev. Drug Discov..

[26] Li G., Yin H., Kuret J. (2004). Casein kinase 1 delta phosphorylates tau and disrupts its binding to microtubules. J. Biol. Chem..

[27] Llorach-Pares L., Nonell-Canals A., Avila C., Sanchez-Martinez M. (2018). Kororamides, convolutamines, and índole derivatives as possible tau and dual-specifity kinase inhibitors for Alzheimer’s disease: A computational study. Mar. Drugs.

[28] Jain P., Karthikeyan C., Moorthy H.N., Waiker D.K., Jain A.K., Trivedi P. (2014). Human CDC2-Like Kinase 1 (CLK1): A Novel Target for Alzheimer’s Disease. Curr. Drug Targets.

[29] Tell V., Hilgeroth A. (2013). Recent developments of protein kinase inhibitors as potential AD therapeutics. Front. Cell Neurosci..

[30] Dolan P.J., Johnson G.V.W. (2010). The role of tau kinases in Alzheimer’s disease. Curr. Opin. Drug Discov. Devel..

[31] Stotani S., Giordanetto F., Medda F. (2016). DYRKlA inhibition as potential treatment for Alzheimer’s disease. Future Med. Chem..

[32] Branca C., Shaw D.M., Belfiore R., Gokhale V., Shaw A.Y., Foley C., Smith B., Hulme C., Dunckley T., Meechoovet B. (2017). Dyrkl inhibition improves Alzheimer’s disease-like pathology. Aging Cell.

[33] Hooper C., Killick R., Lovestone S. (2008). The GSK3 hypothesis of Alzheimer’s disease. J. Neurochem..

[34] Llorens-Martín M., Jurado J., Hemández F., Avila J. (2014). GSK-3, a pivotal kinase in Alzheimer disease. Front. Mol. Neurosci..

[35] Hemández F., Gómez de Barreda E., Fuster-Matanzo A., Lucas J.J., Avila J. (2010). GSK3: A possible link between beta amyloid peptide and tau protein. Exp. Neurol..

[36] Hemandez F., Lucas J.J., Avila J. (2012). GSK3 and tau: Two convergence points in Alzheimer’s disease. J. Alzheimers Dis..

[37] Heneka M.T., Kummer M.P. (2014). Innate immune activation in neurodegenerative disease. Nat. Rev. lmmunol..

[38] Schain M., Kreisl W.C. (2017). Neuroinflammation in neurodegenerative disorders—A review. Curr. Neurol. Neurosci. Rep..

[39] Barbalace M.C., Malaguti M., Giusti L., Lucacchini A., Hrelia S., Angeloni C. (2019). Anti-inflammatory activities of marine algae in neurodegenerative diseases. Int. J. Mol. Sci..

[40] Salter M.W., Stevens B. (2017). Microglia emerge as central players in brain disease. Nat. Med..

[41] Cowan M., Petri W.A. (2018). Microglia: Immune regulators of neurodevelopment. Front. Lmmunol..

[42] Hansen D.V., Hanson J.E., Sheng M. (2018). Microglia in Alzheimer’s disease. J. Cell. Biol..

[43] Colonna M., Butovsky O. (2017). Microglia function in the central nervous system during health and neurodegeneration. Annu. Rev. Immunol..

[44] Dong Y., Li X., Cheng J., Hou L. (2019). Drug development for Alzheimer’s disease: Microglia induced neuroinflammation as a target?. Int. J. Mol. Sci..

[45] Liu C.Y., Wang X., Liu C., Zhang H.L. (2019). Pharmacological targeting of microglial activation: New therapeutic approach. Front. Cell. Neurosci..

[46] Anglister L., Stiles J.R., Salpetert M.M. (1994). Acetylcholinesterase density and turnover number at frog neuromuscular–junctions, with modeling of their role in synaptic function. Neuron.

[47] Ferreira-Vieira T.H., Guimaraes I.M., Silva F.R., Ribeiro F.M. (2016). Alzheimer’s disease: Targeting the cholinergic system. Curr. Neuropharmacol..

[48] Houghton P.J., Ren Y., Howes M. (2006). Acetylcholinesterase inhibitors from plants and fungi. J. Nat. Prod. Rep..

[49] Inestrosa N.C., Alvarez A., Perez C.A., Moreno R.D., Vicente M., Linker C., Casanueva O.I., Soto C., Garrido J. (1996). Acetylcholinesterase accelerates assembly of amyloid-β-peptides into Alzheimer’s fibrils: Possible role of the peripheral site of the enzyme. Neuron.

[50] Alvarez A., Alarcón R., Opazo C., Campos E.O., Muñoz F.J., Calderón F.H., Dajas F., Gentry M.K., Doctor B.P., De Mello F.G. (1998). Stable complexes involving acetylcholinesterase and amyloid-beta peptide change the biochemical properties of the enzyme and increase the neurotoxicity of Alzheimer’s fibrils. J. Neurosci..

[51] Chen J.J., Genereux J.C., Wiseman R.L. (2015). Endoplasmic reticulum quality control and systemic amyloid disease: Impacting protein stability from the inside out. IUBMB Life.

[52] Freitas Silva M., Dias K.S.T., Gontijo V.S., Ortiz C.J.C., Viegas C. (2018). Multi-target directed drugs as a modem approach for drug design towards Alzheimer’s disease: An update. Curr. Med. Chem..

[53] Cummings J., Lee G., Ritter A., Sabbagh M., Zhong K. (2019). Alzheimer’s disease drug development pipeline. Alzheimer’s Dement. Transl. Res. Clin. Interv..

[54] Wang T., Liu X.H., Guan J., Ge S., Wu M.B., Lin J.P. (2019). Advancement of multi-target drug discoveries and promising applications in the field of Alzheimer’s disease. Eur. J. Med. Chem..

[55] Koslow T. (2007). The Silent Deep: The Discovery, Ecology and Consertvation of the Deep Sea.

[56] Russo P., del Bufalo A., Fini A. (2015). Deep sea as a source of novel anticancer drugs: Update on discovery and preclinical/clinical evaluation in a systems medicine perspective. EXCLI J..

[57] Catassi A., Cesario A., Arzani D., Menichini P., Alama A., Bruzzo C., Imperatori A., Rotolo N., Granone P., Russo P. (2006). Characterization of apoptosis induced by marine natural products in non small cell lung cancer A549 cells. Cell Mol. Life Sci..

[58] Russo P., Nastrucci C., Cesario A. (2011). From the sea to anticancer therapy. Curr. Med. Chem..

[59] Nastrucci C., Cesario A., Russo P. (2012). Anticancer drug discovery from the marine environment. Recent Pat. Anticancer Drug Discov..

[60] Russo P., Cesario A. (2012). New anticancer drugs from marine cyanobacteria. Curr. Drug Targets.

[61] Alonso D., Castro A., Martinez A. (2005). Marine compounds for the therapeutic treatment of neurological disorders. Expert Opin. Ther. Pat..

[62] Martins A., Vieira H., Gaspar H., Santos S. (2014). Marketed marine natural products in the pharmaceutical and cosmeceutical industries: Tips for success. Mar. Drugs.

[63] Gerwick W.H., Moore B.S. (2012). Lessons from the past and charting the future of marine natural products drug discovery and chemical biology. Chem. Biol..

[64] Ziegler J., Facchini P.J. (2008). Alkaloids biosynthesis: Methods and trafficking. Annu. Rev. Plant Biol..

[65] Waterman P.G., Roberts M.F., Wink M. (1998). Chemical taxonomy of alkaloids. Alkaloids, Biochemistry, Ecology and Medicinal Applications.

[66] Zhang H., Conte M.M., Khalil Z., Huang X.-C., Capon R.J. (2012). New dictyodendrins as BACE inhibitors from a Southern Australian marine sponge *Ianthella* sp.. RSC Adv..

[67] Zhang H., Conte M.M., Huang X.-C., Khalil Z., Capon R.J. (2012). A search for BACE inhibitors reveals new biosynthetically related pyrrolidones, furanones and pyrroles from a southern Australian marine sponge *Ianthella* sp.. Org. Biomol. Chem..

[68] Meijer L., Skaltsounis A.L., Magiatis P., Polychronopoulos P., Knockaert M., Leost M., Ryan X.P., Vonica C.A., Brivanlou A., Dajani R. (2003). GSK-3-selective inhibitors derived from tyrian purple indirubins. Chem. Biol..

[69] Raot K.V., Doniat M.S., Pengt J., Garcia-Palomero E., Alonso D., Martinez A., Medina M., Franzblau S.G., Tekwanit B.L., Khan S.I. (2006). Manzamine B and E and ircinal A related alkaloids from an indonesian Acanthostrongylophora sponge and their activity against infectious, tropical parasitic, and Alzheimer’s diseases. J. Nat. Prod..

[70] Gompel M., Leost M., de Kier Joffe E.B., Puricelli L., Franco L.H., Puricelli J., Meijer L. (2004). Meridianins, a new family of protein kinase inhibitors isolated from the ascidian Aplidium meridianum. Bioorg. Med. Chem. Lett..

[71] Echalier A.K., Bettayeb K., Ferandin B.Y., Lozach O., Clément M., Valette A., Liger F., Marquet B., Morris J.C., Endicott J.A. (2008). Meriolins (3-(pyrimidin-4-yl)-7-azaindoles): Synthesis, kinase inhibitory activity, cellular effects, and structure of a CDK2/cyclin A/meriolin complex. J. Med. Chem..

[72] Klein-Junior L., Passos C.S., Moraes A., Wakui V., Konrath E., Nurisso A., Carrupt P.-A., Alves de Oliveira C., Kato L., Henriques A. (2014). Indole alkaloids and semisynthetic indole derivatives asmultifunctional scaffolds aiming the inhibition of enzymes related to neurodegenerative diseases—A focus on Psychotria L. Genus. Curr. Top. Med. Chem..

[73] Khanfar M.A., Asal B.A., Mudit M., Kaddoumi A., EI Sayed K.A. (2009). The marine natural-derived inhibitors of glycogen synthase kinase-3 beta- phenylmethylene hydantoins: Ln vitro and in vivo activities and pharmacophore modeling. Bioorg. Med. Chem..

[74] Plisson F., Conte M., Khalil Z., Huang X.-C., Piggott A.M., Capon R.J. (2012). Kinase inhibitor scaffolds against neurodegenerative diseases from a Southern Australian ascidian, *Didemnum* sp.. Chem. Med. Chem..

[75] Alonso E., Vale C., Vieytes M.R., Laferla F.M., Giménez-Llort L., Botana L.M. (2011). 13-Desmethyl spirolide-C is neuroprotective and reduces intracellular Aβ and hyperphosphorylated tau in vitro. Neurochem. Int..

[76] Zhang H., Khalil Z., Conte M.M., Plisson F., Capon R.J. (2012). A search for kinase inhibitors and antibacterial agents: Bromopyrrolo-2- amino-imidazoles from a deep-water Great Australian Bight sponge *Axinella* sp.. Tetrahedron Lett..

[77] Fedorov O., Huber K., Eisenreich A., Filippakopoulos P., King O., Bullock A.N., Szklarczyk D., Jensen L.J., Fabbro D., Trappe J. (2011). Specific CLK inhibitors from a novel chemotype for regulation of alternative splicing. Chem. Biol..

[78] De Souza É.T., Pereira de Lira D., Cavalcanti de Queiroz A., Costa da Silva D.J., Bezerra de Aquino A., Campessato Mella E., Prates Lorenzo V., de Miranda G.E., de Araújo-Júnior J.X., de Oliveira Chaves M.C. (2009). The antinociceptive and anti-inflammatory activities of caulerpin, a bisindole alkaloid isolated from seaweeds of the genus *Caulerpa*. Mar. Drugs.

[79] Kim M.E., Jung I., Na J.Y., Lee Y., Lee J., Lee J.S., Lee J.S. (2018). Pseudane-VII regulates LPS-induced neuroinflammation in brain microglia cells through the inhibition of iNOS expression. Molecules.

[80] Moodie L.W.K., Sepčić K., Turk T., Frangež R., Svenson J. (2019). Natural cholinesterase inhibitors from marine organisms. Nat. Prod. Rep..

[81] Ohlendorf B., Schulz D., Erhard A., Nagel K., Imhoff J.F. (2012). Geranylphenazinediol, an acetylcholinesterase inhibitor produced by a Streptomyces species. J. Nat. Prod..

[82] Kim W.G., Song N.K., Yoo I.D. (2001). Quinolactacins A1 and A2, new acetylcholinesterase inhibitors from *Penicillium citrinum*. J. Antibiot..

[83] Zhang C., Hu L., Liu D., Huang J., Lin W. (2020). Circumdatin D exerts neuroprotective effects by attenuating LPS-induced pro inflammatory responses and downregulating acetylcholinesterase activity in vitro and in vivo. Front. Pharmacol..

[84] Tadokoro Y., Nishikawa T., Ichimori T., Matsunaga S., Fujita M.J., Sakai R. (2017). N-Methyl-beta-carbolinium Salts and an N-Methylated 8-oxoisoguanine as acetylcholinesterase inhibitors from a solitary ascidian, *Cnemidocarpa irene*. ACS Omega.

[85] Kigoshi H., Kanematsu K., Yokota K., Uemura D. (2000). Turbotoxins A and B, novel diiodotyramine derivatives from the Japanese gastropod Turbo marmorata. Tetrahedron.

[86] Tadesse M., Svenson J., Sepčić K., Trembleau L., Engqvist M., Andersen J.H., Jaspars M., Stensvåg K., Haug T. (2014). Isolation and Synthesis of Pulmonarins A and B, acetylcholinesterase inhibitors from the colonial ascidian *Synoicum pulmonaria*. J. Nat. Prod..

[87] Botić T., Defant A., Zanini P., Žužek M.C., Frangež R., Janussen D., Kersken D., Knez Ž., Mancin I., Sepčić K. (2017). Discorhabdin alkaloids from Antarctic *Latrunculia* spp. sponges as a new class of cholinesterase inhibitors. Eur. J. Med. Chem..

[88] Nukoolkarn V.S., Saen-oon S., Rungrotmongkol T., Hannongbua S., Ingkaninan K., Suwanborirux K. (2008). Petrosamine, a potent anticholinesterase pyridoacridine alkaloid from a Thai marine sponge Petrosia n. sp.. Bioorg. Med. Chem..

[89] Moodie L.W., Žužek M.C., Frangež R., Andersen J.H., Hansen E., Olsen E.K., Cergolj M., Sepčić K., Hansen K.Ø., Svenson J. (2016). Synthetic analogs of stryphnusin isolated from the marine sponge *Stryphnus fortis* inhibit acetylcholinesterase with no effect on muscle function or neuromuscular transmission. Org. Biomol. Chem..

[90] Xynas R., Capon R. (1989). 2 Bromotyrosine-derived metabolites from an australian marine sponge *Aplysina* sp.. Aust. J. Chem..

[91] Sepcić K., Marcel V., Klaebe A., Turk T., Suput D., Fournier D. (1998). Inhibition of acetylcholinesterase by an alkylpyridinium polymer from the marine sponge, *Reniera sarai*. Biochim. Biophys. Acta.

[92] Defant A., Mancini I., Raspor L., Guella G., Turk T., Sepcic K. (2011). New structural insights into saraines A, B, and C, macrocyclic alkaloids from the Mediterranean sponge *Reniera (Haliclona) sarai*. Eur. J. Org. Chem..

[93] Orhan I.E., Ozcelik B., Konuklugil B., Putz A., Kaban U.G., Proksch P. (2012). Bioactivity screening of selected Turkish marine sponges and three compounds from *Agelas oroides*. Rec. Nat. Prod..

[94] Kem W.R., Mahnir V.M., Papke R.L., Lingle C.J. (1997). Anabaseine is a potent agonist on muscle and neuronal alphabungarotoxin-sensitive nicotinic receptors. J. Pharmacol. Exp. Ther..

[95] Bharate S.B., Manda S., Joshi P., Singh B., Vishwakarma R.A. (2012). Total synthesis and anti-cholinesterase activity of marine-derived bis-indole alkaloid fascaplysin. Med. Chem. Comm..

[96] Muralidharan A., Josyula V.R., Hariharapura R.C. (2018). Exploring the potential of marine microbes in clinical management of Alzheimer’s disease: A road map for bioprospecting and identifying promising isolates. Life Sci..

[97] Choi D.-Y., Choi H. (2015). Natural products from marine organisms with neuroprotective activity in the experimental models of Alzheimer’s disease, Parkinson’s disease and ischemic brain stroke: Their molecular targets and action mechanisms. Arch. Pharm. Res..

[98] Rao K.V., Kasanah N., Wahyuono S., Tekwani B.L., Schinazi R.F., Hamann M.T. (2004). Three new manzamine alkaloids from a common Indonesian sponge and their activity against infectious and tropical parasitic diseases. J. Nat. Prod..

[99] Yousaf M., Hammond N.L., Peng J., Wahyuono S., McIntosh K.A., Charman W.N., Mayer A.M.S., Hamann M.T. (2004). New manzamine alkaloids from an indo-pacific sponge. Pharmacokinetics, oral availability, and the significant activity of several manzamines against HIV-I, AIDS opportunistic infections, and inflammatory diseases. J. Med. Chem..

[100] Peng J., Hu J.F., Kazi A.B., Li Z., Avery M., Peraud O., Hill R.T., Franzblau S.G., Zhang F., Schinazi R.F. (2003). Manadomanzamines A and B: A novel alkaloid ring system with potent activity against mycobacteria and HIV-1. J. Am. Chem. Soc..

[101] Bharate S.B., Yadav R.R., Battula S., Vishwakarma R.A. (2012). Meridianins: Marine-derived potent kinase inhibitors. Mini Rev. Med. Chem..

[102] Franco L.H., Joffe E.B.D.K., Puricelli L., Tatian M., Seldes A.M., Palermo J.A. (1998). Indole alkaloids from the tunicate *Aplidium meridianum*. J. Nat. Prod..

[103] Lebar M.D., Baker B.J. (2010). Synthesis and structure reassessment of psammopemmin A. Aust. J. Chem..

[104] Giraud F., Alves G., Debiton E., Nauton L., Théry V., Durieu E., Ferandin Y., Lozach O., Meijer L., Anizon F. (2011). Synthesis, protein kinase inhibitory potencies, and in vitro antiproliferative activities of meridianin derivatives. J. Med. Chem..

[105] Tahtouh T., Elkins J.M., Filippakopoulos P., Soundararajan M., Burgy G., Durieu E., Cochet C., Schmid R.S., Lo D.C., Delhommel F. (2012). Selectivity, cocrystal structures, and neuroprotective properties of leucettines, a family of protein kinase inhibitors derived from the marine sponge alkaloid leucettamine B. J. Med. Chem..

[106] Perry N.B., Ettouati L., Litaudon M., Blunt J.W., Munro M.H.G., Parkin S., Hope H. (1994). Alkaloids from the Antarctic sponge *Kirkpatrickia varialosa*. Part 1: Variolin B, a new antitumour and antiviral compound. Tetrahedron.

[107] Carroll A.R., Wild S.J., Duffy S., Avery V.M. (2012). Kororamide A, a new tribrominated índole alkaloid from australian bryozoan *Amathia tortuosa*. Tetrahedron Lett..

[108] Dashti Y., Vial M.L., Wood S.A., Mellick G.D., Roullier C., Quinn R.J. (2015). Korforamide B, a brominated alkloid from the bryozoan *Amathia tortuosa* and its effects on Parkinson’s disease cells. Tetrahedron.

[109] Gul W., Hamann M.T. (2005). Indole alkaloid marine natural products: An established source of cancer drug leads with considerable promise for the control of parasitic, neurological and other diseases. Life Sci..

[110] Ciavatta M.L., Lefranc F., Vieira L.M., Kiss R., Carbone M., van Otterlo W.A.L., Lopanik N.B., Waeschenbach A. (2020). The *phylum* bryozoa: From biology to biomedical potential. Mar. Drugs.

[111] Kang H., Fenical W. (1997). Ningalins A-D: Novel aromatic alkaloids from a Western Australian ascidian of the genus *Didemnum*. J. Org. Chem..

[112] Falk M., Burton I.W., Hu T., Walter J.A., Wright J.L.C. (2001). Assignment of the relative stereochemistry of the spirolides, macrocyclic toxins isolated from shellfish and from the cultured dinoflagellate *Alexandrium ostenfeldii*. Tetrahedron.

[113] Supriyono A., Schwarz B., Wray V., Witte L., Müller W.E., van Soest R., Sumaryono W., Proksch P. (1995). Bioactive alkaloids from the tropical marine sponge *Axinella carteri*. Z. Für Nat. C.

[114] Meijer L., Thunnissen A.M.W.H., White A.W., Garnier M., Nikolic M., Tsai L.H., Walter J., Cleverley K.E., Salinas P.C., Wu Y.Z. (2000). Inhibition of cyclin-dependent kinases, GSK3β and CK1 by hymenialdisine, a marine sponge constituent. Chem. Biol..

[115] Sharma G.M., Buyer S.S., Pomerantz M.W. (1980). Characterization of a yellow compound isolated from the marine sponge *Phakellia flabellate*. J. Chem. Soc. Chem. Comm..

[116] Lindel T., Jensen P.R., Penicai W., Long B.H., Casazza A.M., Carboni J., Pairchild C.R. (1997). Eleutherobin, a new cytotoxin that mimics paclitaxel (Taxol) by stabilizing microtubules. J. Am. Chem. Soc..

[117] Long B., Carboni J.M., Wasserman A.J., Comell L.A., Casazza A.M., Jensen P.R., Lindel T., Fenical W., Fairchild C.R. (1998). Eleutherobin, a novel cytotoxic agent that induces tubulin polymerization, is similar to paclitaxel (Taxol (R)). Cancer Res..

[118] D’Ambrosio M., Guerriero A., Pietra P., Sarcodictyin A., Sarcodictyin B. (1987). Novel diterpenoidic alcohols esterified by (E)-N(l)-methylurocanic acid. Isolation from the Mediterranean stolonifer *Sarcodictyon roseum*. Helv. Chim. Acta.

[119] D’Anbrosio M., Guerriero A., Pietra P. (1988). lsolation from the Mediterranean stolonifern coral *Sarcodictyon roseum* of sarcodictyin C, D, E, and P, novel diterpenoidic alcohols esterified by (E)- or (Z)-N(1)-methylurocanic acid. Failure of the carbon-skeleton type as a classification criterion. Helv. Chim. Acta..

[120] Ciomei M., Albanese C., Pastori W., Grandi M., Pietra P., D’Ambrosio M., Guerriero A., Battistini C. (1997). Sarcodictyins: A new class of marine derivatives with mode of action similar to Taxol. Abstract 30. Proc. Am. Ass. Canc. Res..

[121] Barbosa M., Valentão P., Andrade P.B. (2014). Bioactive Compounds from Macroalgae in the New Millennium: Implications for Neurodegenerative Diseases. Mar. Drugs..

[122] Kim M.E., Jung I., Lee J.S., Na J.Y., Kim W.J., Kim Y.O., Park Y.D. (2017). Pseudane-VII isolated from *Pseudoalteromonas* sp. M2 ameliorates LPS-induced inflammatory response in vitro and in vivo. Mar. Drugs..

[123] Li J.L., Huang L., Liu J., Song Y., Gao J., Jung J.H., Liu Y., Chen G. (2015). Acetylcholinesterase inhibitory dimeric indole derivatives from the marine actinomycetes *Rubrobacter radiotolerans*. Fitoterapia.

[124] Sangnoi Y., Sakulkeo O., Yuenyongsawad S., Kanjana-Opas A., Ingkaninan K., Plubrukarn A., Suwanborirux K. (2008). Acetylcholinesterase-inhibiting activity of pyrrole derivatives from a novel marine gliding bacterium, *Rapidithrix thailandica*. Mar. Drugs.

[125] Beedessee G., Ramanjooloo A., Surnam-Boodhun R., van Soest R.W.M., Marie D.E.P. (2013). Acethylcholinesterase-inhibitory activities of the extracts from sponges collected in Mauritius waters. Chem. Biodivers..

[126] Proksch P., Ebel R., Edrada R., Riebe F., Liu H., Diesel A., Bayer M., Li X., Lin W.H., Grebenyuk V. (2008). Sponge-associated fungi and their bioactive compounds: The Suberites case. Bot. Mar..

[127] Cheng Z.Q., Song J.L., Zhu K., Zhang J., Jiang C.S., Zhang H. (2018). Total synthesis of pulmonarin B and design of brominated phenylacetic acid/tacrine hybrids: Marine pharmacophore inspired discovery of neu Che and Aβ aggregation inhibitors. Mar. Drugs.

[128] Yang A., Baker B.J., Grimwade J., Leonard A., McClintock J.B. (1995). Discorhabdin alkaloids from the Antarctic sponge *Latrunculia apicalis*. J. Nat. Prod..

[129] Antunes E.M., Beukes D.R., Kelly M., Samaai T., Barrows L.R., Marshall K.M., Sincich C., Davies-Coleman M.T. (2004). Cytotoxic pyrroloiminoquinones from four new species of south African *latrunculide* sponges. J. Nat. Prod..

[130] Perry N.B., Blunt J.W., Munro M.H. (1988). Cytotoxic pigments from New Zealand sponges of the genus *Latrunculia*: Discorhabdin-A, Discorhabdin-B and Discorhabdin-C. Tetrahedron.

[131] Reyes F., Martín R., Rueda A., Fernández R., Montalvo D., Gómez C., Sánchez-Puelles J.M. (2004). Discorhabdins I and L, cytotoxic alkaloids from the sponge *Latrunculia brevis*. J. Nat. Prod..

[132] Lidgren G., Bohlin L., Bergman J. (1986). Studies of Swedish marine organisms 7. A novel biologically active indole alkaloid from the sponge *Geodia baretti*. Tetrahedron Lett..

[133] Sölter S., Dieckmann R., Blumenberg M., Francke W. (2002). Barettin, revisited?. Tetrahedron Lett..

[134] Olatunji O.J., Ogundajo A.L., Oladosu I.A., Changwichit K., Ingkanina K., Yuenyongsawad S., Plubrukarn A. (2014). Non-competitive inhibition of acetylcholinesterase by bromotyrosine alkaloids. Nat. Prod. Commun..

[135] Tabudravu J.N., Jaspars M. (2002). Purealidin S and purpuramine J, bromotyrosine alkaloids from the Fijian marine sponge *Druinella* sp.. J. Nat. Prod..

[136] Jurek J., Yoshida W.Y., Scheuer P.J., Kelly-Borges M. (1993). 3 New bromotyrosine-derived metabolites of the sponge *Psammaplysilla purpurea*. J. Nat. Prod..

[137] Sepčić K., Mancin I., Vidic I., Franssanito R., Pietra F., Macek P., Turk T. (2001). Antibacterial and anticholinesterase activities of aplysamine-4, a bromotyrosine-derived metabolite of a Red Sea marine sponge. J. Nat. Toxins.

[138] Moody K., Thomson R., Fattorusso E., Minale L., Sodano G. (1972). Aerothionin and homoaerothionin—2 tetrabromo spirocyclohexadienylisoxazoles from *Verongia* sponges. JCS Perkin I..

[139] Sirimangkalakitti N., Olatunji O.J., Changwichit K., Saesong T., Chamni S., Chanvorachote P., Ingkaninan K., Plubrukarn A., Suwanborirux K. (2015). Bromotyrosine alkaloids with acetylcholinesterase inhibitory activity from the Thai sponge *Acanthodendrilla* sp.. Nat. Prod. Commun..

[140] Gopichand Y., Schmitz F. (1979). Marine natural-products—Fistularin-1, fistularin 2 and fistularin-3 from the sponge *Aplysina fistularis* forma *fluva*. Tetrahedron Lett..

[141] Cimino G., de Rosa S., de Stefano S., Sodano G. (1986). Marine natural products: New results from Mediterranean invertebrates. Pure Appl. Chem..

[142] Cimino G., de Stefano S., Scognamiglio G., Sodano G., Trivellone E. (1986). Sarains A new class of alkaloids from the marine sponge *Reniera sarai*. Bull. Soc. Chim. Belg..

[143] Cimino G., Mattia C.A., Mazzarella L., Puliti R., Scognamiglio G., Spinella A., Trivellone E. (1989). Unprecedented alkaloid skeleton from the Mediterranean sponge *Reniera sarai*: X-ray structure of an acetate derivative of sarain-A. Tetrahedron.

[144] Cimino G., Spinella A., Trivellone E. (1989). Isosarain-1: A new alkaloid from the Mediterranean sponge *Reniera sarai*. Tetrahedron Lett..

[145] Guo Y., Madaio E., Trivellone E., Scognamiglio G., Cimino G. (1996). Further studies of alkaloids from *Reniera sarai*: Structures of saraine-3 and isosaraine-3, absolute stereochemistry of saraine-1 and saraine-2. Tetrahedron.

[146] Guo Y., Trivellone E., Scognamiglio G., Cimino G. (1998). Absolute stereochemistry of isosaraine-1 and isosaraine-2. Tetrahedron Lett..

[147] Langjae R., Bussarawit S., Yuenyongsawad S., Ingkaninan K., Plubrukarn A. (2007). Acetylcholinesterase-inhibiting steroidal alkaloid from the sponge *Corticium* sp.. Steroids.

[148] Forenza S., Minale L., Riccio R., Fattorusso E. (1971). New bromo-pyrrole derivatives from the sponge *Agelas oroides*. J. Chem. Soc. D Chem. Commun..

[149] Garcia E.E., Benjamin L.E., Fryer R.I. (1973). Reinvestigation into the structure of oroidin, a bromopyrrole derivative from a marine sponge. J. Chem. Soc. Chem. Commun..

[150] Turk T., Macek P., Suput D. (1995). Inhibition of acetylcholinesterase by a pseudozoanthoxanthin-like compound isolated from the zoanthid *Parazoanthus axinellae* (O. Schmidt). Toxicon.

[151] Šuput J.S., Turk T., Maček P., Šuput D. (1996). Pseudozo-anthoxantin-like compound from *Parazoanthus axinellae* Adriaticus inhibits acetylcholinesterase. Pflugers Arch..

[152] Schwartz R.E., Yunker M.B., Scheuer P.J., Ottersen T. (1979). Pseudo-zoanthoxanthins from gold coral. Can. J. Chem..

[153] Vitale R.M., Rispoli V., Desiderio D., Sgammato R., Thellung S., Canale C., Vassalli M., Carbone M., Ciavatta M.L., Mollo E. (2018). In silico identification and experimental validation of novel anti-Alzheimer’s multitargeted ligands from a marine source featuring a “2-aminoimidazole plus aromatic group” scaffold. ACS Chem. Neurosci..

[154] Albizati K.F., Faulkner D.J. (1985). Stevensine, a novel alkaloid of an unidentified sponge. J. Org. Chem..

[155] Coates R.M., Kem W.R., Abbott B.C. (1971). Isolation and structure of a hoplonemertine toxin. Toxicon.

[156] Papke R.L., Meyer E.M., Lavieri S., Bollampally S.R., Papke T.A.S., Horenstein N.A., Itoh Y., Papke J.K.P. (2004). Effects at a distance in α7 nAChR selective agonists: Benzylidene substitutions that regulate potency and efficacy. Neuropharmacology.

[157] Wheeler J.W., Olubajo O., Storm C.B., Duffield R.M. (1981). Anabaseine: Venom alkaloid of Aphaenogaster ants. Science.

[158] Bourguet-Kondracki M.-L., Kornprobst J.M. (2005). Marine pharmacology: Potentialities in the treatment of infectious diseases, osteoporosis and Alzheimer’s disease. Adv. Biochem. Engin/Biotechnol..

[159] Hentschel J., Piel S., Degnan M., Taylor M.W. (2012). Genomic insights into the marine sponge microbiome. Nat. Rev. Microbiol..

[160] Manda S., Sharma S., Wani A., Josi P., Kumar V., Guru S.K., Bharate S.S., Bhushan S., Vishwakarma R.A., Kumar A. (2016). Discovery of a marine-derived bis-indole alkaloid fascaplysin, as a new class of potent P-glycoprotein inducer and establishement of its structure–activity relationship. Eur. J. Med. Chem..

[161] Sun Q., Liu F., Sang J., Lin M., Ma J., Xiao X., Yan S., Naman C.B., Wang N., He S. (2019). 9-Methyl -fascaplysin is a more potent Aβ aggregation inhibitor than the marine-derived alkaloid, fascaplysin, and produces nanomolar neuroprotective effects in SH-SY5Y cells. Mar. Drugs.

[162] Pan H., Qiu H., Zhang K., Zhang P., Liang W., Yang M., Mou C., Lin M., He M., Xiao X. (2019). Fascaplysin derivatives are potent multitarget agents against Alzheimer’s disease: In vitro and in vivo evidence. ACS Chem. Neurosci..

[163] Blunt J.W., Copp B.R., Keyzers R.A., Munro M.H., Prinsep M.R. (2015). Marine natural products. Nat. Prod. Rep..

[164] Cavalli A., Bolognesi M.L., Minarini A., Rosini M., Tumiatti V., Recanatini M., Melchiorre C. (2008). Multi-target-directed ligands to combat neurodeghenerative diseases. J. Med. Chem..

[165] Zhou J., Jiang X., He S., Jiang H., Feng F., Liu W., Qu W., Sun H. (2019). Rational design of multitarget-directed ligands: Strategies and emerging paradigms. J. Med. Chem..

[166] Prati F., Uliassi E., Bolognesi M. (2014). Two disease, one approach: Multitarget drug discovery in Alzheimer’s and neglected tropical diseases. Med. Chem. Comm..

